# Specialized cytonemes induce self-organization of stem cells

**DOI:** 10.1073/pnas.1920837117

**Published:** 2020-03-17

**Authors:** Sergi Junyent, Clare L. Garcin, James L. A. Szczerkowski, Tung-Jui Trieu, Joshua Reeves, Shukry J. Habib

**Affiliations:** ^a^Centre for Stem Cells and Regenerative Medicine, King’s College London, SE1 9RT London, United Kingdom

**Keywords:** development, cell signaling, tissue formation, cell–cell communication, stem cell

## Abstract

Spatial cellular organization is fundamental for embryogenesis. Remarkably, coculturing embryonic stem cells (ESCs) and trophoblast stem cells (TSCs) recapitulates this process, forming embryo-like structures. However, mechanisms driving ESC–TSC interaction remain elusive. We describe specialized ESC-generated cytonemes that react to TSC-secreted Wnts. Cytoneme formation and length are controlled by actin, intracellular calcium stores, and components of the Wnt pathway. ESC cytonemes select self-renewal–promoting Wnts via crosstalk between Wnt receptors, activation of ionotropic glutamate receptors (iGluRs), and localized calcium transients. This crosstalk orchestrates Wnt signaling, ESC polarization, ESC–TSC pairing, and consequently synthetic embryogenesis. Our results uncover ESC–TSC contact–mediated signaling, reminiscent of the glutamatergic neuronal synapse, inducing spatial self-organization and embryonic cell specification.

## Significance

Many questions about how stem cells communicate with neighboring cells and self-organize to initiate tissue formation remain unanswered. We uncovered mechanisms employed by embryonic stem cells (ESCs) and trophoblast stem cells (TSCs) to coform embryo-like structures. We describe ESC-generated cytonemes that react to self-renewal–promoting Wnt ligands secreted by TSCs. We identified glutamatergic activity upon formation of ESC–TSC interaction. This cellular connection is required for the transmission of Wnt signals to ESCs for Wnt/β-catenin pathway activation, a process that regulates morphogenesis. Given that many stem cell types express glutamate receptors and rely on niche-secreted Wnt ligands for self-renewal, we propose that Wnt and glutamatergic signaling crosstalk may prove prevalent in various mammalian tissues to regulate stem cell–niche interactions.

Stem cells reside in cellular niches that impart chemical and physical signals to regulate their self-renewal and differentiation (1). Understanding the communication between the niche and stem cells paved the way to engineering organoids that mimic in vivo tissue structures. Organoids are invaluable to the study of development and tissue patterning, for drug screening, and for regenerative medicine applications. Synthetic embryos that resemble the blastocyst (2) and the gastrulating embryo (3, 4) have recently been described. These structures offer unique insights into processes of morphogenesis and patterning that can be challenging to study in the naturally developing embryo. To generate synthetic embryos, embryonic stem cells (ESCs) and trophoblast stem cells (TSCs) are mixed and allowed to self-sort and organize to develop the embryonic structure.

Wnt/β-catenin signaling has been implicated in tissue patterning and the self-renewal of many types of mammalian stem cells, including embryonic stem cells (5). This pathway is regulated by Wnt ligands, which are often secreted locally from the stem cell niche (6). Wnt proteins bind to the low-density lipoprotein-related receptors 5 and 6 (LRP5/6) and to a member of the Frizzled (Fzd) receptor family. Ligand binding induces phosphorylation of the cytoplasmic tail of LRP6, the binding of Disheveled protein (DVL) to the cytoplasmic domain of Fzd, and the inhibition of the destruction complex that targets β-catenin for degradation. Consequently, β-catenin is stabilized, translocates to the nucleus, and initiates the Wnt-mediated transcription program. In the context of many mammalian stem cells, this program blocks differentiation and promotes self-renewal (7).

The secretion of Wnt ligands must be spatially and temporally controlled to provide local cues that regulate the abundance of tissue stem cells and the differentiation of progeny cells once exiting the niche (8). To produce position-dependent information during a specific time frame, diffusible morphogenetic gradients are a seemingly implausible mechanism because they lack precision and temporal dynamics (9). Conversely, contact-dependent signaling ensures the transfer of the developmental signal via direct cell–cell contact (10). In 1999, Ramírez-Weber and Kornberg identified cytonemes as signaling filopodia that orient toward morphogen-producing cells and specialize in recruiting developmental signals (11). Others have also identified cytonemes made by ligand-producing cells, which extend the ligand toward responsive cells (12–14). Both cytoneme types limit ligand dispersion and effectively facilitate ligand delivery to the responding cells. Cytonemes exist in *Drosophila* tissues and in other organisms, including Zebrafish (15), vertebrate embryos (16), and cultured human cells (17).

Mechanisms that regulate ESC–TSC communication and their spatial organization to generate synthetic embryos are incompletely defined. Additionally, knowledge of how mammalian stem cells distinguish and receive niche signals to facilitate their division and determine cell fate remains elusive. To address these issues, we followed the interaction between ESCs and TSCs at single-cell resolution. We found that ESCs extend cytonemes that can contact TSCs and recognize secreted Wnts, resulting in ESC–TSC pairing. When Wnt ligand secretion in TSCs was inhibited, ESC–TSC pairing and consequently the formation of synthetic embryos significantly decreased.

We investigated whether the cytonemes of ESCs distinguish between Wnt ligands that activate the Wnt/β-catenin pathway (e.g., Wnt3a) versus other Wnts that transduce β-catenin–independent pathways (e.g., Wnt5a). Therefore, we immobilized purified Wnt3a and Wnt5a onto microbeads, distributed the microbeads around single ESCs, and investigated the interaction between cytonemes and Wnt beads. Our results indicate that ESCs can distinguish between signals and selectively reinforce a connection to the self-renewal Wnt3a ligand in an LRP6-dependent process. This signal recruitment is also mediated by the activity of α-amino-3-hydroxy-5-methyl-4-isoxazolepropionic acid (AMPA)/kainate glutamate receptors at the cytonemes, which produces calcium transients. We identified the roles of intracellular calcium stores, Wnt receptors, DVL2, and β-catenin in regulating the formation and length of ESC cytonemes.

In conclusion, we demonstrate that ESCs possess specialized cytonemes that react to self-renewal signals and orchestrate ESC–TSC pairing, setting the basis for spatial organization and specification of embryonic tissues.

## ESCs Extend Cytonemes to Initiate Contact with TSCs

**Fig. 1.**
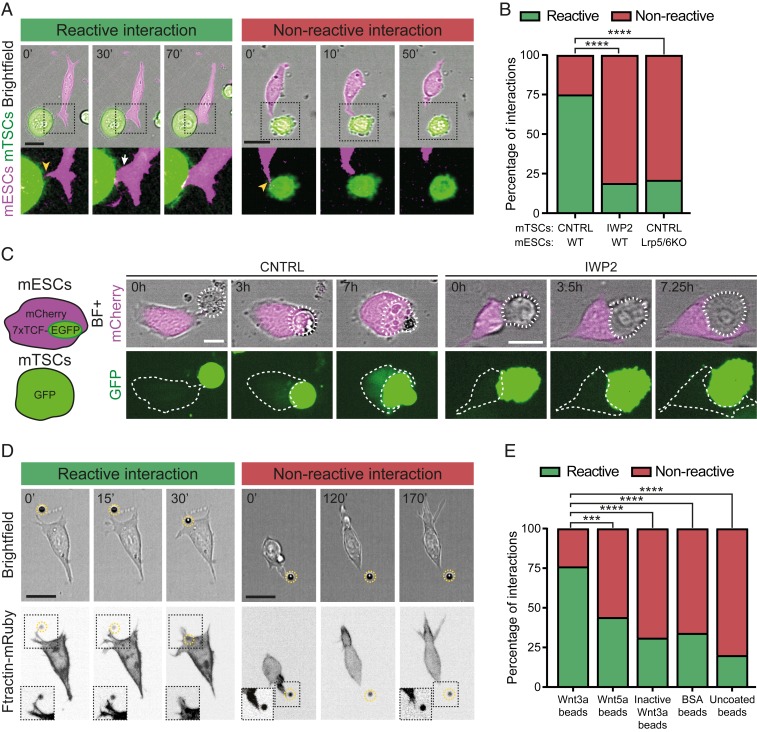
ESCs selectively react to self-renewal–promoting Wnt signals and initiate pairing with TSCs. (*A*) Representative frames from time-lapse imaging of ESCs (expressing Ftractin-mRuby3; magenta) interacting with TSCs that express eGFP (green). Examples of reactive interactions (*Left*, green) and nonreactive interactions (*Right*, red) are shown. (Scale bars, 20 μm.) Time is expressed in minutes. Arrowheads (yellow) indicate initial interaction through thin protrusions; the arrow (white) indicates larger protrusion. (*B*) Quantification of the percentage of reactive (green) and nonreactive (red) interactions between ESCs and TSCs in different conditions. *n* ≥ 44 from more than three independent experiments. (*C*) Schematic (*Left*) and representative images (*Right*) of ESCs expressing the 7xTCF-EGFP//simian virus 40 early promoter (SV40)-mCherry (a Wnt/β-catenin pathway reporter) and contacting TSCs. Shown are representative images of EGFP expression levels in ESCs contacting WT cells (CNTRL) or 24-h IWP2-pretreated TSCs. In both conditions, 100 ng/mL R-spondin was added to the media to increase pathway activation. Dashed white line marks TSCs (*Top*) and ESCs (*Bottom*). (Scale bars, 20 µm.) Time is expressed in hours. (*D*) Representative images from time-lapse imaging of ESCs expressing the F-actin reporter Ftractin-mRuby3 (*Bottom*, grayscale) and interacting with beads. Reactive (*Left*, green) and nonreactive (*Right*, red) interactions are shown. (Scale bars, 20 μm.) Time is expressed in minutes. *Insets* are magnified and contrast-enhanced for clarity. (*E*) Quantification of the percentage of reactive (green) and nonreactive (red) interactions between ESCs and different types of beads. *n* ≥ 41 cells from at least three independent experiments. Asterisks indicate statistical significance calculated by Fisher’s exact test: ***P < 0.001; ****P < 0.0001.

ESCs and TSCs possess the ability to self-sort and organize when cultured together to generate embryonic structures (2–4). By time-lapse imaging, we investigated how the initial interaction between cell types was achieved. Single TSCs, which constitutively expressed enhanced green fluorescent protein (eGFP), displayed limited movement (Fig. 1*A*). We used ESCs expressing the F-actin reporter Ftractin-mRuby (18), permitting visualization of fine membranous structures during ESC–TSC interactions. We observed single ESCs extending protrusions that transiently contacted TSCs. After the initial contact, ESCs reacted by directing a larger protrusion to establish a stable contact with TSCs (reactive interaction; RI), often followed by ESC–TSC pairing (Fig. 1*A* and Movie S1). We did not observe TSCs contacting ESCs in a similar manner to establish ESC–TSC pairing.

ESCs rely on activation of the Wnt/β-catenin pathway for self-renewal (19, 20). Therefore, we investigated whether TSCs secrete Wnt ligands that are received by ESCs. We profiled the transcripts of the 19 Wnt genes in TSCs, showing the expression of 16 Wnt transcripts (*SI Appendix*, Fig. S1*A*). Importantly, the interaction of ESCs and TSCs can result in activation of the Wnt/β-catenin pathway in ESCs, as indicated by the 7 oligomerized T cell factor (TCF)-binding sites (7xTCF)-eGFP (21) ESC reporter line (Fig. 1*C* and *SI Appendix*, Fig. S1*B*). To verify if the TSCs were, in fact, the source of the Wnt ligands, we opted to use a short-term inhibition of Wnt ligand secretion to minimize the potential impact on TSC maintenance and identity. Accordingly, ESCs incubated with TSCs pretreated with inhibitor of Wnt production-2 (IWP2), a small molecule that blocks the secretion of Wnt ligands (22), for 24 h significantly reduced the magnitude of activation, similar to that of ESCs cultured alone (Fig. 1*C* and *SI Appendix*, Fig. S1*B*). These results indicate that TSCs produce Wnt ligands that are received by ESCs to activate the Wnt/β-catenin pathway.

Next, we determined whether the ESC–TSC interaction itself is affected by IWP2 treatment of TSCs. We observed that ESCs contact treated TSCs transiently via the protrusions; however, in 76% of cases this was not followed by ESC–TSC pairing (nonreactive interaction; Fig. 1 *A* and *B* and Movie S2). We obtained similar results using a different Wnt secretion inhibitor, Wnt-C59 (ref. 23, Fig. S1C). We speculated that the ESC protrusions are cytonemes that sense TSC-derived Wnt ligands, which are essential for the establishment of stable contacts during ESC–TSC pairing. To confirm this, we generated a double knock-out (dKO) of the Wnt coreceptors LRP5 and LRP6 in ESCs (LRP5/6dKO) and observed that the transient contact between cytonemes and TSCs was unaffected. However, these ESCs had a significantly reduced ability to establish stable contacts with TSCs, similarly to the ESC interaction with IWP2-pretreated TSCs (Fig. 1*B*). Furthermore, both IWP2 (or Wnt-C59) treatment and LRP5/6dKO ESCs resulted in a significant reduction in the formation of synthetic embryo structures (3) in three-dimensional (3D) culture (*SI Appendix*, Fig. S2). Our results suggest that specialized ESC cytonemes induce ESC–TSC pairing, an essential step in synthetic embryogenesis.

To study the specificity of these cytonemes for Wnt ligands, we covalently immobilized purified Wnts to microbeads and investigated the cytoneme-bead interactions.

## ESCs Selectively Recruit Wnt Ligands Required for Self-Renewal

**Fig. 2.**
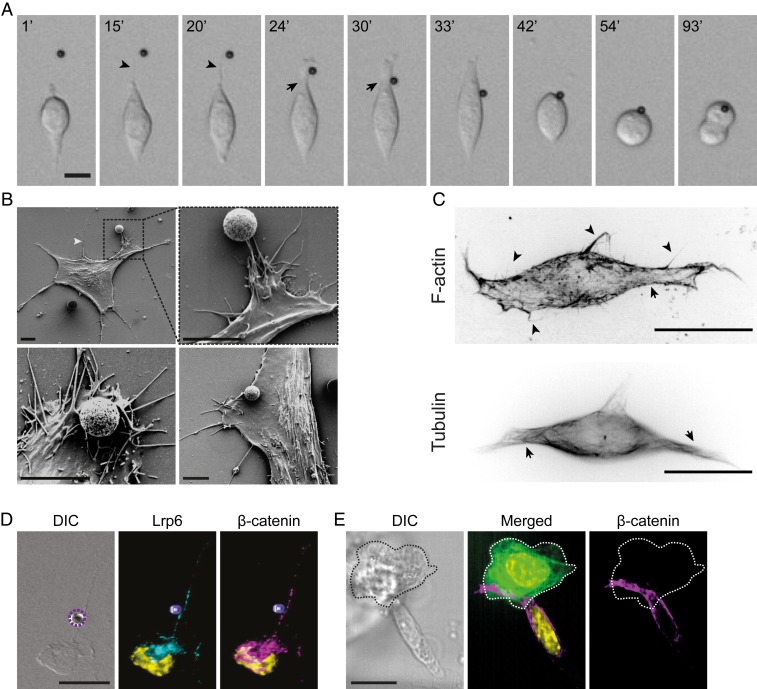
ESCs extend exploratory actin-based cytonemes that recruit localized Wnt signals to the plasma membrane. (*A*) Representative frames from time-lapse imaging of an ESC contacting a Wnt3a bead (black sphere) with a cytoneme. Time is expressed in minutes. (Scale bar, 10 μm.) Arrowheads indicate thin cytonemes; arrows indicate larger cytonemes used for Wnt-bead recruitment. (*B*) Scanning electron microscopy images of ESCs at various stages of interaction with the Wnt3a bead (white/gray spheres). White arrowhead indicates a thin cytoneme. (Scale bar, 5 μm.) (*C*) Deconvolved structural illumination images of ESCs stained with the live-cell cytoskeleton-staining reagents SiR-Actin (*Top*) and SiR-Tubulin (*Bottom*). Arrows indicate larger cytonemes; arrowheads indicate thin cytonemes. (Scale bars, 10 μm.) (*D*) Representative images of an ESC cytoneme contacting a Wnt3a bead (dashed purple circle or 3D-reconstructed purple sphere) stained with antibodies against LRP6 (cyan) and β-catenin (magenta) with DAPI (yellow). (Scale bar, 20 μm.) (*E*) Representative images of an ESC contacting an eGFP-expressing TSC (green, dashed edge) stained with antibodies against β-catenin (magenta) with DAPI (yellow). (Scale bar, 20 μm.)

We previously described a system of a localized Wnt3a bead which recapitulates a niche signal essential for self-renewal and oriented asymmetric cell division (ACD) of single ESCs (20). Wnt5a, also produced by TSCs, cannot activate the Wnt/β-catenin pathway in ESCs (20). Importantly, Wnt5a beads do not induce ACD in ESCs (20). Using this Wnt-bead approach, we aimed to investigate the mechanisms by which ESCs interact with localized niche signals. We incubated single cells in close proximity to Wnt3a beads or Wnt5a beads (*SI Appendix*, Fig. S1*D*) and monitored initial cell-bead contact by live imaging. Primary observations revealed that ESCs utilize thin cytonemes to contact the bead and can react by directing a larger cytoneme to recruit the bead to the plasma membrane (reactive interaction [RI]) to form a stable contact (Figs. 1*D* and 2*A* and Movie S3). Although Wnt5a has high protein sequence similarity to Wnt3a, our assay indicated a significantly higher proportion of reactive interactions when cytonemes encountered Wnt3a beads (76% RI) relative to Wnt5a beads (43% RI) (Fig. 1*E*). This suggests that ESC cytonemes selectively react to Wnt ligands required for self-renewal.

We also tested the reactivity of cytonemes to control beads—inactive Wnt3a beads (iWnt3a beads) treated with dithiothreitol (DTT) to break the disulfide bridges in Wnt ligands, thus disrupting protein tertiary structure to render it inactive (refs. 20 and 24 and *SI Appendix*, Fig. S1*D*)—or to uncoated beads. Our results indicate that the cytonemes are unable to react to iWnt3a beads or uncoated beads efficiently (31% and 20% RI, respectively; Fig. 1*E*). To further confirm the selectivity of the cytonemes, we exposed ESCs to beads coated with bovine serum albumin (BSA), a nonsignaling molecule that often adheres nonspecifically to cellular membranes. Here, only 34% of interactions were reactive (Fig. 1*E*), reduced like the other control beads.

In summary, ESCs generate ligand-selective cytonemes to identify and recruit Wnt signals required for self-renewal. This ligand-based selectivity also governs the efficiency of stable ESC–TSC contacts and pairing.

To determine how cytonemes achieve this dual functionality, we analyzed their composition and dynamics.

## ESCs Produce Actin-Based Cytonemes that Contain Components of the Wnt Signaling Pathway

Single ESCs appear to predominantly present bimodal, elongated cytonemes that emanate from the cell body (Fig. 2*A* and Movie S4), although more cytonemes can form subsequently (Figs. 2*B* and 4 *B* and *C* and *SI Appendix*, Fig. S3*A*). Cytonemes are dynamic and retract upon cell rounding prior to division (Fig. 2*A*). Cytonemes can form on different substrates and in culture media that support the self-renewal of single ESCs (*SI Appendix*, Fig. S3 *A* and *B*). These cytonemes are also present in ESC lines with different genetic backgrounds (*SI Appendix*, Fig. S3*C*). The time required to generate cytonemes varies between different ESC lines, but after five hours the percentage of cells with cytonemes is similar (*SI Appendix*, Fig. S3*D*). Scanning electron microscopy (SEM) revealed two major cytoneme types that both appear capable of interacting with the Wnt3a bead: thin cytonemes (∼100-nm length) emanating from the main body of the cell and other nanostructures that are generated from larger cytonemes (Fig. 2*B*).

To further characterize ESC cytonemes, we investigated their molecular composition. All observed cytonemes are composed mainly of actin, with tubulin restricted to the large cytonemes (Fig. 2*C*). Inhibition of actin polymerization via Cytochalasin D (*SI Appendix*, Fig. S4 *A*–*C*) prevented the formation of new cytonemes and restricted the motility of both the cell and the existing cytonemes (*SI Appendix*, Fig. S4*C*). Blockade of actin filament bundling using the Fascin inhibitor (Fascin-G2) also prevented cytoneme formation (*SI Appendix*, Fig. S4*D*). Therefore, cells without bundled actin filaments lose the capacity to recruit beads. Conversely, inhibition of tubulin polymerization by Colcemid did not impact this process (*SI Appendix*, Fig. S4 *E*–*J*). Cytonemes contain components of the Wnt signaling pathway. On average, 58.9% of the observed cytonemes (*n* = 39 of analyzed single ESCs) contain LRP6 and all cytonemes have the downstream signaling component β-catenin (*SI Appendix*, Fig. S4 *K* and *L*). Contact with the Wnt source (Wnt3a bead or TSC) leads to the polarization of the Wnt coreceptor LRP6 and β-catenin in the ESC toward the area of contact (Fig. 2 *D* and *E*).

In summary, to selectively recruit the self-renewal signal Wnt3a, ESCs produce actin-based cytonemes that contain receptors and downstream effectors of the Wnt signaling pathway. Cytoneme-Wnt3a bead contact polarizes the bulk of the Wnt signaling components to the Wnt source.

## ESC Cytonemes Activate Ionotropic Glutamate Receptors in Response to a Self-Renewal–Promoting Wnt Source

**Fig. 3.**
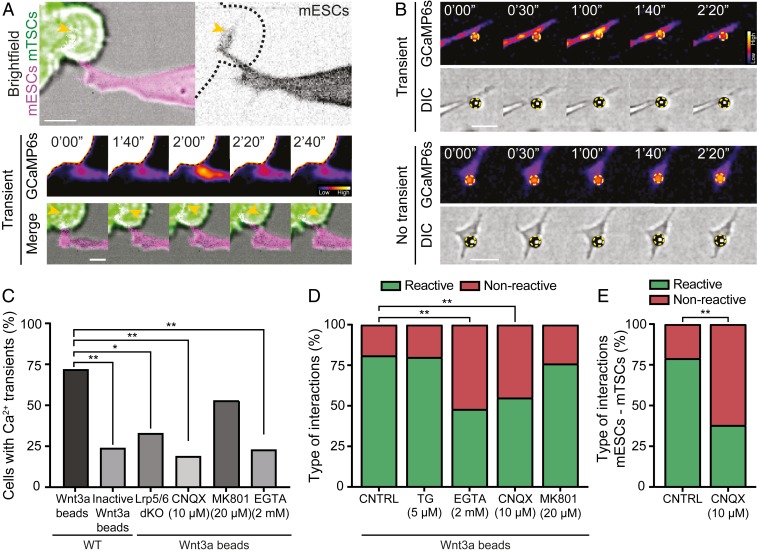
ESC cytonemes exhibit glutamate receptor activity upon contact with a Wnt source. (*A*) Representative frames from time-lapse imaging of an ESC (*Left*, magenta; *Right*, grayscale) expressing the Ca^2+^ reporter GCaMP6s and Ftractin-mRuby3, where a cytoneme contacts an eGFP-expressing TSC (green, dashed black line). (*Top*) interaction between ESC and TSCs, (Scale bar, 10 μm); (*Middle*) generation of Ca^2+^ transients upon cytoneme-TSC contact; with fluorescence signal represented by heat-map colors using the Fire look-up table in Fiji; (*Bottom*) position of the cytoneme in the interaction (yellow arrowhead points to the tip of the cytoneme). Time is expressed in minutes and seconds. (Scale bar, 5 μm.) (*B*) Representative frames from time-lapse imaging of an ESC expressing GCaMP6s, where a cytoneme contacts a Wnt3a bead. (*Top*) generation of Ca^2+^ transients upon cytoneme−Wnt3a bead contact; (*Bottom)* absence of Ca^2+^ transients, with the bead shown as a black sphere in the DIC panels, outlined by a yellow dashed circle. Time is expressed in minutes and seconds. (Scale bars, 10 μm.) DIC, differential interference contrast. (*C*) Quantification of the percentage of cells with Ca^2+^ transients in the cytonemes, in different ESC cell lines, in the presence of Wnt3a or iWnt3a beads or cells treated with CNQX (10 μM), MK801 (20 μM), or EGTA (2 mM). (*D*) Quantification of the percentage of reactive (green) or nonreactive (red) interactions between ESCs and Wnt3a beads in the presence of 5-μM TG, 2-mM EGTA, 10-μM CNQX, 20-μM MK801, or control treatment water or dimethyl sulfoxide (DMSO; CNTRL). Water or DMSO treatment yielded similar percentages of interactions. *n* ≥ 30 cells from three independent experiments. (*E*) Quantification of the percentage of reactive (green) or nonreactive (red) interactions between ESCs and TSCs in the presence of 10-μM CNQX. *n* = 55 cells from three independent experiments. Asterisks indicate statistical significance calculated by Fisher’s exact test for *C*, *D*, and *E*: ns, not significant; *P < 0.05; **P < 0.01; ***P < 0.001; ****P < 0.0001.

Contact-mediated signaling shares similarities with the neuronal synapse (25). In both systems, cellular protrusions are extended to receive a paracrine signal from the producing/presynaptic cell. In the neuronal synapse, calcium (Ca^2+^) influx is often involved in neurotransmission and Ca^2+^ channels can be found on the pre- and postsynapse (26). We examined the generation of Ca^2+^ transients at the cytonemes of ESCs upon contact with a Wnt source. To do this, we generated a stable cell line expressing the free-cytoplasmic Ca^2+^ sensor GCaMP6s (27) and employed live imaging every six seconds (Fig. 3 *A* and *B*). A stable contact between an ESC and a TSC generated localized Ca^2+^ transients on the ESC cytoneme (Fig. 3*A*). Similarly, upon a stable contact between a Wnt3a bead and the ESC cytoneme, 72% of the cells generated localized Ca^2+^ transients at the area of contact (Fig. 3 *B* and *C*). Only 24% of the cells had these transients when contacting an iWnt3a bead (Fig. 3 *B* and *C*). Importantly, the Wnt3a beads induced more frequent and longer-duration Ca^2+^ transients than the iWnt3a beads (*SI Appendix*, Fig. S5*A*).

Chelating Ca^2+^ from the media by ethylene glycol tetraacetic acid (EGTA) significantly reduced Ca^2+^ transients (only 25% of the cells) upon a stable contact between a Wnt3a bead and the cytoneme (Fig. 3*C* and *SI Appendix*, Fig. S5*A*). These results suggest that Ca^2+^ influx is mediated by activated Ca^2+^ protein channels/transporters at the Wnt3a bead contact area. We sought to investigate those protein channels or transporters.

Huang et al. (28) recently demonstrated the generation of Ca^2+^ transients at the cytonemes of *Drosophila* air sac primordium that are essential for Decapentaplegic (Dpp) signaling. These Ca^2+^ transients require glutamatergic activity mediated by the glutamate receptor GluRII. Crosstalk between ionotropic glutamate receptors, Ca^2+^ influx, and Wnt signaling has also been described in the neuronal synapse (29). We aimed to investigate whether this feature also exists at the ESC cytoneme. Transcript profiling (*SI Appendix*, Fig. S5*B*) and proteomic analysis indicate that ESCs express ionotropic glutamate receptors (iGluRs) (30, 31). Initially, we incubated single ESCs with Wnt3a beads in the presence of inhibitors of iGluRs: MK801 (a noncompetitive antagonist of the *N*-methyl-D-aspartate [NMDA] receptor) or cyanquixaline (CNQX), a competitive antagonist for the AMPA and kainate receptors. Only CNQX treatment significantly reduced the proportion of cells that produce Ca^2+^ transients at the cytonemes upon a stable contact with a Wnt3a bead (19%) (Fig. 3*C*). Here, the transient frequency was low and each lasted only 21.8 s, on average, relative to 78.2 s without CNQX (*SI Appendix*, Fig. S5*A*). Consequently, CNQX treatment significantly reduced the proportion of reactive interactions with Wnt3a beads, thus impacting Wnt3a-bead recruitment to the plasma membrane (Fig. 3*D*). The agonist kainate can reverse the effects of CNQX on Ca^2+^ transients and the reactivity of the cytonemes to Wnt3a beads (*SI Appendix*, Fig. S5 *C* and *D*). Importantly, the proportion of ESC cytonemes that establish a stable contact with TSCs and mediate ESC–TSC pairing, as well as the proportion of synthetic embryo structures (3) that can form in 3D, was significantly reduced in the presence of CNQX (Fig. 3*E* and *SI Appendix*, Fig. S2).

Next, we asked if the activity of iGluRs, intracellular Ca^2+^, and Ca^2+^ influx are required for cytoneme formation. CNQX and MK801 had no significant effect on cytoneme formation (*SI Appendix*, Fig. S5 *E* and *F*). Depleting intracellular Ca^2+^ stores by Thapsigargin (TG, a SERCA pump blocker) (32, 33) or 1,2-Bis(2-aminophenoxy)ethane-*N*,*N*,*N*,*N*-tetraacetic acid tetrakis(acetoxymethyl ester) (a cell-permeable Ca^2+^ chelator) (34) significantly compromised cytoneme formation (*SI Appendix*, Fig. S5*E*). However, Thapsigargin treatment did not affect the ability of the existing cytonemes to react to Wnt3a beads (Fig. 3*D*). On the other hand, EGTA in the media significantly reduced the proportion of reactive cytonemes (Fig. 3*D*). This supports the role of iGluR-mediated signaling, which includes Ca^2+^ influx to the cell, in the recruitment of localized Wnt3a to cells.

In conclusion, the formation of ESC cytonemes is regulated by intracellular calcium stores. Activated AMPA/kainate receptors enable localized influxes of Ca^2+^ on cytonemes that facilitate the recruitment of the self-renewal signal Wnt and mediate ESC–TSC pairing and the formation of synthetic embryos.

## Components of the Wnt Pathway Regulate the Formation and Selectivity of Cytonemes

The distribution patterns of Wnt receptors and downstream effectors such as β-catenin within cytonemes prior to cell-signal contact suggests that ESCs may utilize these proteins to regulate reactivity to an instructive signal. To examine this, we generated knock-out (KO) cell lines and studied the effect of the loss of each component on cytoneme formation and the recruitment of Wnt3a beads. Specifically, we generated LRP5KO, LRP6KO, LRP5/6dKO, and DVL2KO and utilized the published conditional β-catenin KO cell line (βKO) (35).

We initially characterized these cell lines including the expression of pluripotency markers, the protein levels of components of the Wnt/β-catenin pathway, and the ability of the different KO cell lines to activate the Wnt/β-catenin pathway. Please refer to *SI Appendix*, *Supplementary Text* and Figs. S6 and S7 for a full description of the generation and characterization of the KO cell lines.

Briefly, when cultured in 2i media that maintain ESC self-renewal (36), KO cell lines of the receptors and DVL2 maintain levels of pluripotency markers comparable to those of wild-type (WT) cells (*SI Appendix*, Fig. S6). Importantly, in this media CHIR 99021 (a glycogen synthase kinase 3 β [GSK-β] inhibitor) can activate the Wnt/β-catenin signaling pathway by inhibiting the destruction complex, overriding the need for Wnt proteins, the receptors, or DVL2 (19). The freshly knocked-out β-catenin cell line has reduced levels of pluripotency markers compared to the WT cell line (*SI Appendix*, Fig. S7).

**Fig. 4.**
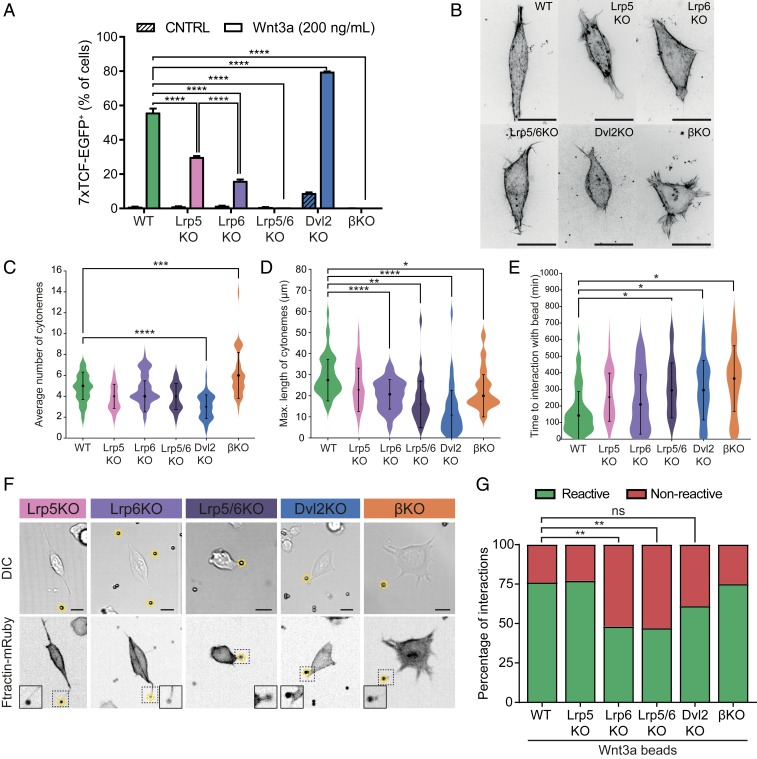
Components of the Wnt-pathway regulate the formation and dynamics of the cytonemes of ESCs. (*A*) Response of the 7xTCF-eGFP Wnt/β-catenin pathway reporter in WT, LRP5KO, LRP6KO, LRP5/6KO, DVL2KO, and βKO treated with media containing either 200 ng mL^−1^ soluble Wnt3a or vehicle for 24 h, as measured by fluorescent activated cell sorting (FACS) flow cytometry profile. Asterisks indicate statistical significance as calculated by two-way ANOVA test. (*B*) Representative deconvolved structural illumination microscopy images of WT and KO ESCs stained with live-cell cytoskeleton staining reagent SiR-Actin. (Scale bar, 20 μm.) (*C* and *D*) Violin plots of the distribution and average number of cytonemes per cell (*C*) and the distribution and average maximum length of a cytoneme per cell normalized to the average cell radius (*D*) for WT and KO ESCs. (*E*) Violin plots of the distribution and average time necessary for bead contact in WT or KO ESCs. For *C*–*E*, *n* > 40 cells from three independent experiments. Asterisks indicate statistical significance calculated by one-way ANOVA tests. For detailed statistical analysis, see *SI Appendix*, Fig. S8 *B*–*D*. (*F*) Representative images of the different KO ESC lines, stably expressing the F-actin reporter Ftractin-mRuby and contacting Wnt3a beads with a nano-cytoneme. Wnt3a beads are indicated by yellow dashed circles. (Scale bars, 10 μm.) (*G*) Quantification of reactive (green) or nonreactive (red) interactions between WT or KO ESCs and Wnt3a beads. *n* ≥ 39 from three independent experiments. Asterisks indicate statistical significance calculated by Fisher’s exact test. For all panels, asterisks indicate statistical significance as: ns, not significant; *P < 0.05; **P < 0.01; ***P < 0.001; ****P < 0.0001.

In the presence of purified Wnt3a ligands, leukemia inhibitory factor and serum-containing media LRP5KO has a significant reduction in activation of the Wnt/β-catenin pathway in comparison to WT, and LRP6KO is significantly further compromised. LRP5/6dKO cannot activate the Wnt/β-catenin pathway in this media. Furthermore, DVL2KO, which has elevated levels of DVL1 and DVL3, can activate the Wnt/β-catenin pathway even more than the WT cells in the presence of Wnt3a ligands (Fig. 4*A*).

We next addressed whether the aforementioned differences between WT and mutant cells impact cytoneme formation and reactivity to niche signals. WT cells elongate and mainly produce bimodal cytonemes, but they can also have additional smaller cytonemes. Combined, these cytonemes, averaging five per single WT cell with an average maximum length of 29.9 μm, facilitate interaction with the surrounding Wnt3a beads (Fig. 4 *B*–*D* and Movie S4). Similarly, the cell lines LRP5KO, LRP6KO, and LRP5/6dKO have, on average, four cytonemes. LRP5KO cytonemes are marginally shorter when compared to WT cytonemes (Fig. 4 *B*–*D*, *SI Appendix*, Fig. S8 *A*–*C*, and Movie S5). On the other hand, LRP6KO has significantly shorter cytonemes (21.1 μm) than WT and the cytonemes are even shorter in the LRP5/6dKO cells (16.3 μm) (Fig. 4 *B*–*D*, *SI Appendix*, Fig. S8 *A*–*C*, and Movie S6). DVL2KO cells are rounder than WT cells and contain significantly fewer cytonemes (an average of three cytonemes) that are very short (10 μm) in comparison to all other studied cell lines (Fig. 4 *B*–*D*, *SI Appendix*, Fig. S8 *A*–*C*, and Movie S7). The round morphology is more prominent in βKO (Fig. 4*B*), although βKO has more (an average of seven) and shorter cytonemes than the WT cells (Fig. 4 *B*–*D*, *SI Appendix*, Fig. S8 *A*–*C*, and Movie S8). The aforementioned statistically significant changes in the number and length of the protrusions of each KO cell line (Fig. 4 *C* and *D*) are reflected in the increased time required by these cells to detect Wnt3a beads in their local environment (Fig. 4*E* and *SI Appendix*, Fig. S8*D*). Importantly, in these measurements the initial distance between the cell and the bead was similar for all cell lines (*SI Appendix*, Fig. S8*E*).

All KO cell lines establish interactions with Wnt3a beads via cytonemes (Fig. 4*F*). Knocking out LRP6 significantly reduces the ability of a cell to subsequently recruit the Wnt3a bead to the plasma membrane, whereas knocking out LRP5 does not (Fig. 4*G*). LRP5/6dKO also exhibits compromised ability to recruit Wnt3a beads to the cell and to pair with TSCs (Figs. 4*G* and 1 *A* and *B*). We found that LRP5/6dKO also impaired the generation of Ca^2+^ transients upon cytoneme−Wnt3a bead contact (only 36% of the cells; Fig. 3*C*). The frequency and duration of the Ca^2+^ transients observed at LRP5/6dKO cytonemes were reduced in comparison to WT (*SI Appendix*, Fig. S5*A*). These results emphasize the essential crosstalk between Wnt coreceptors and Ca^2+^ transients at the cytonemes for the recruitment of localized Wnt signals and ESC–TSC pairing.

The interactions of DVL2KO cytonemes are less reactive than those of the WT, whereas the reactivity of the βKO cytoneme interactions is unaffected (Fig. 4*G*).

Taken together, the evidence shows that LRP6 plays an important role in the initial recognition and subsequent recruitment of the Wnt3a bead to the plasma membrane after contact, while LRP5 is not required for this process. DVL2 and β-catenin regulate the length and number of cytonemes and the resulting efficiency of detecting the self-renewal signal without compromising its recruitment to the plasma membrane.

In summary, we identified specialized ESC-produced cytonemes and the mechanism they use to detect and selectively react to self-renewal Wnts. This process requires crosstalk between Wnts and the family of glutamate receptors that involves an influx of Ca^2+^ and promotes ESC–TSC pairing required for synthetic embryogenesis.

## Discussion

Cytoneme-mediated signaling is a means of highly specific paracrine signal transduction, allowing both signal amplitude and duration to be controlled with exquisite precision. Yamashita and colleagues identified, in male germline stem cells, microtubule-based protrusions that contain bone morphogenic protein (BMP) receptors and extend to the hub cells in *Drosophila* testis, thereby recruiting the ligand that is essential for their maintenance (37). Identification of cytonemes and understanding of their function in mammalian stem cells remain limited.

Here, we show that ESCs use specialized cytonemes to distinguish between niche signals and preferentially select Wnt ligands promoting their self-renewal, such as those secreted by TSCs. After the recognition of the Wnt source, ESCs generate larger cytonemes to facilitate robust signaling and pairing with TSCs to promote synthetic embryogenesis.

The multiplicity of secreted ligands and the difficulty of visualizing Wnt proteins in situ make it challenging to study how Wnt reception occurs in ESCs. To circumvent these issues, we utilized a reductionist approach of immobilizing purified Wnt ligands on microbeads, distributing them near single ESCs, and observing their interaction by time-lapse imaging.

We found that ESCs generate actin-based cytonemes that contain Wnt receptors, which represent, on average, 60% of the total protrusions that ESCs form. After a Wnt source is detected, the bulk of the receptors and β-catenin polarizes toward the Wnt. A larger cytoneme enriched with Wnt receptors and AMPA/kainate receptor subunits form a stable contact with the Wnt source. Consequently, LRP6-AMPA/kainate receptor crosstalk is initiated and generates localized Ca^2+^ transients. This crosstalk is required to allow the reactive interaction, Wnt signaling, and ESC–TSC pairing that form the basis of cellular communication and spatial self-organization.

The finding that ESCs utilize iGluR-containing cytonemes indicates a striking similarity to aspects of the neuronal synapse. Both systems show selective, directed cellular protrusions for the purposes of reinforcing a “correct” signal and leading to higher-order spatial signaling and organization. Recent comprehensive investigations have demonstrated that components of the pre- and postsynapse are essential for cytoneme-mediated paracrine signaling in the *Drosophila* air sac primordium (28) and that neurotransmitter pathway components, including glutamatergic receptors, are present in Ctenophore during embryogenesis (38). These findings, and our own data, may suggest that the neuronal synapse shares a common ancestor with glutamatergic cytonemes used for cell–cell signaling, illustrating an evolutionarily conserved method for spatial organization of cells.

Intracellular Ca^2+^ stores and components of the Wnt pathway regulate cytoneme formation. Specifically, components of the Wnt pathway control the number of cytonemes, their length, and their selectivity to the ligand. LRP6, but not LRP5, regulates the length of the cytoneme. This may be attributed to motifs in the N terminal of LRP6 that can bind to regulators of actin filaments and promote their elongation, branching, and dynamics (39–41). Similarly, DVL2 has been shown to interact with actin regulators (42). DVL2KO produces fewer and shorter cytonemes in comparison to all other studied cell lines. We found that, as a Wnt-pathway effector, β-catenin levels are inversely correlated with the number of cytonemes. While the absence of β-catenin leads to an increase in the number of cytonemes, higher levels of β-catenin, such as those reported in Dvl2KO, repress cytoneme formation and consequently produce fewer and shorter cytonemes than WT. Considering all evidence, we showed that Wnt pathway components in ESCs not only are required for signal reception and transduction but also determine the efficiency of scanning the environment to locate self-renewal signals.

In summary, we identified specialized cytonemes that form the basis of ESC–TSC communication that is fundamental to morphogenesis. The minimal approach of single ESCs interacting with immobilized niche ligands provides unique insights into the mechanisms of contact-mediated signaling. We envision that mechanisms identified in this study could be applied to adult stem cells and niches where the Wnt/β-catenin pathway is required for self-renewal.

## Materials and Methods

Details of the protocols for culture of ESCs and TSCs, generation and characterization of knockout ESC and reporter cell lines, purification and immobilization of Wnt proteins, conditions of cell imaging, image analysis, electron microscopy, flow cytometry, and statistical analyses performed can be found in *SI Appendix*.

### Data and Materials Availability.

All additional data and information are included in *SI Appendix* as figures, additional legends, and references.

## Supplementary Material

Supplementary File

Supplementary File

Supplementary File

Supplementary File

Supplementary File

Supplementary File

Supplementary File

Supplementary File

Supplementary File
