# Supplementary material for: Specialized cytonemes induce self-organization of stem cells
Source: Proc Natl Acad Sci U S A. 2020 Mar 17;117(13):7236–44. doi: 10.1073/pnas.1920837117 (PMC7132109; doi:10.1073/pnas.1920837117)
Supplement: Supplementary File [file pnas.1920837117.sapp.pdf]

PNAS
www.pnas.org

Supplementary Information for

Specialized cytonemes induce self-organization of stem cells

Sergi Junyent, Clare L. Garcin*, James L.A. Szczerkowski*, Tung-Jui Trieu*, Joshua Reeves, and Shukry J. Habib

Shukry J. Habib
Email: shukry.habib@kcl.ac.uk

This PDF file includes:

Supplementary Materials and Methods
Figures S1 to S8
Legends for Movies 1 to 8
Supplementary text
SI References

Other supplementary materials for this manuscript include the following:

Movies 1 to 8

*C.L.G., J.L.A.S., and T.-J.T. contributed equally to this work

www.pnas.org/cgi/doi/10.1073/pnas.1920837117

Supplementary Materials and Methods

Cell culture

Wild-type (W4 129S6/SvEvTac, referred as WT in the text), heterozygous β-catenin deficient ($\beta^{f/-}$), with an inducible floxed β-catenin allele, and stably carrying a Cre-ER-T2 cassette under the control of a chicken β-actin promoter (1), or long-term β-catenin deficient ($\beta^{-/-}$) (**Fig. S7A**) mouse embryonic stem cell (ESC) lines were maintained in ESC complete media (Advanced DMEM/F12, Life Technologies, #12634028 or phenol red free DMEM/F12, Life Technologies, #21041-025), containing 10% ES qualified foetal bovine serum (FBS, Millipore #ES-009-B), 1% penicillin-streptomycin (P-S, Sigma, #P4333), 2 mM Glutamax (Life Technologies, 35050061) and 50 µM β-mercaptoethanol (2ME, Gibco, #21985-023), containing 1000 U/mL Leukemia Inhibitory Factor (LIF; Miltenyi, #130-095-775). For cell maintenance, ESC media was supplemented with 2i: MEK inhibitor PD0325901 (Miltenyi, #130-104-170) (1 µM) and GSK3 inhibitor CHIR99021 (3 µM) (Miltenyi, #130-104-172). Cells were grown until formation of mid-sized colonies, passaged every 3-4 days, and the media was changed every other day unless stated otherwise. In some experiments, 24 hours prior to use, 2i was withdrawn and replaced with soluble Wnt3a protein (200 ng/mL). Generation of inducible β-catenin knock-out from $\beta^{f/-}$ cells was achieved through the addition of 0.1 µg/mL 4-hydroxy-tamoxifen (4OHT) (Sigma, #H6278) to the media every 24 hours for 72 hours (referred as βKO or $\beta^{f/-}$ +3d 4OHT in the text).

Trophoblast stem cells (TSCs) expressing eGFP (derived by the Rossant Lab (2) were cultured on a layer of mitotically inactivated mouse embryonic fibroblasts (MEFs), as described before (3). Briefly, MEFs where cultured in tissue culture-treated dishes to confluency in MEF media (DMEM supplemented with 10% FBS, 100 µM 2ME, 2 mM Glutamax, 1% P-S). MEFs were mitotically inactivated by treatment with 10 µg/mL Mitomycin C (Sigma, #M0503) for 2h at 37°C, 5%CO_2, and thoroughly rinsed with PBS before further use. TSCs were cultured in mitotically inactivated MEFs in TSF4H media (RPMI1640 supplemented with 20% FBS, 100 µM 2ME, 2 mM Glutamax, 1% P-S, 1 mM sodium pyruvate (Life technologies), 25 ng/mL FGF4 (R&D technologies, #5846-F4) and 1 µg/mL Heparin (Sigma, #H3393). Media was changed daily, and colonies were split as required. 24h before experiment, TSCs were weaned from MEFs; cells were trypsinized (0.05% Trypsin-EDTA), centrifuged (4 min, 1,000 x *g*) and resuspended in TSF4H media. To remove MEFs, cells were transferred twice to a clean tissue culture-treated plate, and MEFs were allowed to attach for 15 min at 37°C, 5%CO_2. TSCs in the supernatant were then transferred to a clean culture plate and incubated for 24h in TSF4H-conditioned media (70% TSF4H-MEF conditioned media for 3 days plus 30% fresh TSF4H media, with a final concentration of 25 ng/mL FGF4 and 1 µg/mL Heparin). In some experiments 2 µM IWP2 (Miltenyi, #130-105-335) or 1 µM Wnt-C59 (Cayman Chemicals, #16644) was added to the media (indicated in the text).

Cells were regularly checked for mycoplasma infection.

Plasmids

pLV-Ftractin-mRuby3-p2A-mTurquoise-MLC-IRES-Blast was a gift from Tobias Meyer (Addgene plasmid #85146). XE131 XFz1-CS2P+ was a gift from Randall Moon (Addgene plasmid #16793). pGP-CMV-GCaMP6s was a gift from Douglas Kim (Addgene plasmid #40753). pSpCas9(BB)-2A-GFP (PX458) was a gift from Feng Zhang (Addgene plasmid #48138). 7xTCF-eGFP/SV40-mCherry (4) and the lentiviral packaging plasmids pCMVΔR8.74 and pMD2.VSVG were a gift from Roel Nusse.

Transfection/Lentiviral Infection

For xFzd1-GFP transfection, 8 x10^4 ESCs were seeded into a 12-well plate well plate, and incubated overnight at 37°C, 5%CO_2. 2 μg plasmid was transfected using JetPEI (Polyplus transfection, #101-10). Cells were incubated overnight, and construct expression was verified prior use in the experiments.

Lentiviruses were produced in HEK 293T cells. 5 x10^6 HEK cells were seeded into 10 cm^2 dishes 24 hours prior to transfection. Cells were transfected using JetPEI with 10 μg lentiviral vector, 3.5 μg VSV-G envelope plasmid (pMD2.VSVG), and 6.5 μg of the packaging plasmid (pCMVΔR8.74) in a final volume of 500 μL 150 mM NaCl. Media was replaced after 16 hours and conditioned twice for 24 hours. Conditioned media was collected and filtered (0.45 μm PES filter, Millipore, #SLHP033RS) and concentrated using Lenti-X concentrator (overnight at 4°C) (Clontech, #631232) before centrifuging 1500 x *g* for 45 minutes and the pellet resuspended in media containing FBS. Virus were stored at -80°C until use.

Wnt purification and immobilization

Recombinant Wnt3a proteins were produced in Drosophila S2 cells grown in suspension in Schneider's Drosophila Medium (Life Technologies) and Wnt3a proteins were purified according to the protocol described by Willert (5). Conditioned media was collected and filtered, and Wnt3a protein was purified. Initially, conditioned media was applied to a Blue Sepharose Column to recover the majority of the Wnt3a proteins from the conditioned media. Conditioned media was passed over the column at a constant flow rate. Following application of the conditioned media, the column was washed with Binding Buffer (1% (w/v) CHAPS, 150 mM KCl, 20 mM Tris-HCl, pH 7.5, sterile filtered). Protein was then eluted by the addition of elution buffer to the column: 1% (w/v) CHAPS, 1.5 M KCl, 20 mM Tris-HCl, pH 7.5, sterile filtered. Eluted liquid was collected as fractions, which were all tested for the presence of Wnt proteins via Western blotting. Wnt3a activity was tested via superTOPFlash reporter assay: stably transfected L cells (LS/L cells) cultured in DMEM containing 10% FBS and 1% P-S were exposed to soluble Wnt3a proteins for no longer than 15

hours before cell lysates were collected. Wnt-induced luciferase activity was determined via the Dual-Light System (Applied Biosystems) and Luciferase readings were taken on a Glomax (Promega). Wnt3a protein and Wnt5a protein were also purchased from R&D Systems (#1324-WN, #645-WN).

Wnt proteins were immobilized onto 2.8 µm Dynabeads®, and their biological activity was tested as described elsewhere (6). Briefly, the carboxylic acid groups on 2.8 µm Dynabeads® M-270 Carboxylic Acid (Invitrogen, #14305D) were activated by 30-minute incubation with carbodiimide (Sigma, E7750-1G) and N-hydroxyl succinamide (Sigma, #56480-25G) (50 mg/mL each, dissolved in 25 mM cold 2-(N-morpholino) ethanesulfonic acid (MES) (Sigma, #M3671-50G) buffer (pH5) with constant rotation. Following activation, beads were retained within the tube through the use of a magnet and washed three times with 25 mM MES buffer (pH5). Soluble Wnt3a or Wnt5a protein (500 ng) was diluted 1:5 in MES buffer (pH5) and incubated with the beads for 1 hour with constant agitation. For the generation of BSA beads, activated beads where incubated with 0.1% BSA-PBS solution at room temperature for 1 hour with constant agitation. Beads were washed again three times with PBS (pH 7.4) before storage in media containing 10% FBS at 4°C. Inactivation of Wnt3a beads was achieved through incubation with 10 mM Dithiothreitol (DTT; Life Technologies, #P2325) for 30 minutes at 37°C. Following incubation with DTT, beads were washed three times in PBS before storage in media containing 10% FBS at 4°C (up to 10 days).

Bead activity was tested as described above for soluble Wnt. Briefly, an appropriate concentration of beads (usually 2 µg/well) were added to pre-seeded 50,000 LS/L cells and incubated for 15 hours before the signal was compared as a fold change to inactive beads, soluble Wnt, and a vehicle control. For Wnt5a beads, the activity was measured as their capacity to compete with soluble Wnt3a in an LSL assay (6) (**Fig. S1D**).

<u>Live cell imaging</u>

ESCs were seeded at 2500 cells + 0.3 µg Wnt3a beads or other beads per well of a clear-bottomed black 96 well plate in complete media containing LIF. The 96 well plate was placed into the Zeiss inverted Axio Imager epifluorescence microscope, equipped with a CoolSNAP HQ2 CCD camera, and cells were allowed to settle in the microscope at 37°C, 5% CO_2 for 30 minutes prior to imaging. Between 20-30 positions were selected, and cells imaged at 10X (N/A = 0.3) in brightfield every 1 minute for 12-15 hours. Videos were analyzed in FIJI to measure the type, number, maximum length and dynamics of the cytonemes. For this purpose, single cells that attached, generated cytonemes and contacted a bead were selected. The time elapsed between attachment and bead contact was registered, and the number and length of the cytonemes was measured at ten timepoints over that period. Where the inhibitors CytochalasinD (CytoD, ThermoFisher,

#PHZ1063), Colcemid (Deme-colcine, Sigma, #D-7385) or Fascin-G2 (Xcessbio, #M60269) were used, cells were pre-seeded for 4 hours in a well of a clear -bottomed black 96 well plate prior to addition of a range of CytoD, Colcemid or Fascin-G2 concentrations diluted in ESC media. Alternatively, cells where trypsinized, centrifuged and resuspended in ESC media containing CytoD, Colcemide or Fascin-G2 at the concentration stated in the figures. DMSO and H_2O were used as carrier controls for CytoD or Fascin-G2 and Colcemid respectively. Cells were imaged in a Zeiss inverted Axio Imager epifluorescence microscope, at 10X (N/A = 0.3) in brightfield every 15 minutes for 4 hours. Time-lapse movies were analysed in FIJI to measure the percentage of cells with cytonemes, as well as the dynamics of the cytonemes, when applied. In some experiments, BAPTA-AM (10 μM, Sigma, #A1076), Thapsigargin (5 μM, Sigma, #T9033), CNQX (10 μM, Sigma, #C127), (+)MK801 (20 μM, Sigma, #M107) and EGTA (2 mM, Sigma, E0396) were added to the media during imaging.

To analyze the behavior of the ESC cytonemes, ESCs expressing the fluorescently labeled xFzd1-GFP or the F-actin reporter Ftractin-mRuby3 were seeded at 2500 cells + 0.3 μg beads per well in a clear-bottomed black 96 well plate, in complete ESC media containing LIF. To analyse ESC-TSC interaction, 3,000 ESCs and 3,000 TSCs were seeded together, in ESC media. The plates were placed into the Nikon Eclipse Ti Inverted Spinning Disk confocal (equipped with a Yokogawa CSU-1 disk head and an Andor Neo sCMOS camera), and cells were allowed to settle in the microscope at 37°C, 5% CO2 for 30 minutes prior imaging. Between 15 to 25 positions were selected, laser intensity was adjusted as required, three z-positions were defined, and cells were imaged in DIC and the corresponding fluorescent channel every 10-15 minutes for 12 hours using a 20x objective. We visualized protrusion interaction with beads tethered to ligands or with TSCs. Reactive and non-reactive interactions were classified as described in **Fig. 1**. In some experiments, Kainate (100 μM, FisherScientific, #15467999), Thapsigargin, CNQX, (+)MK801, EGTA, IWP2 or Wnt-C59 were added to the media during imaging.

To analyse Wnt pathway activation upon ESC-TSC contact, 3,500 ESC and 3,500 TSC cells were seeded on a clear-bottomed 96-well plate in ESC media containing LIF. The imaging was set-up in the Inverted Spinning Disk confocal as described. Images were acquired every 15 minutes for 24h using a 20x objective. For all experiments, images were analyzed using FIJI.

Live-cell staining of tubulin and actin was achieved by pre-staining ESCs with 1 μM SiR-tubulin or SiR-actin containing ESCs media + LIF for 1h. Cells were then trypsinized, neutralized, and resuspended on 100 nM SiR-Tubulin or SiR-actin containing ESC media + LIF. Cells where counted and seeded at single cell density and were allowed to settle and generate cytonemes for 4 hours

prior imaging using the Nikon Eclipse Ti-2 Inverted iSIM (structural illumination microscope, equipped with Hamamatsu Flash4.0 imaging system).

Fluorescence activated cell sorting

Fluorescence activated cell sorting (FACS) was employed to detect Wnt3a response of the studied ESCs stably infected with 7xTCF-eGFP//SV40-mCherry. Infected cells were sorted to gain a pure mCherry^{+} population. Following stimulation with Wnt3a or control media (ESC complete media + LIF), cells were harvested using 0.25% trypsin-EDTA solution, filtered and analyzed using the FACSFortessa system (BD Biosciences). The gating strategy included gating for SSC-FSC, SSC-A-SSC-W, SSC-DAPI (alive cells), SSC-PE Texas Red (mCherry^{+} cells), and SSC-FITC (GFP^{+} cells). To analyze Wnt pathway activation upon ESC-TSC interaction, ESC expressing 7xTCF-eGFP/SV40-mCherry cells where cultured for 24h in contact with control-treated TSCs or TSCs pre-treated for 24h with IWP2 (2 µM). In some conditions 100 ng/mL R-spondin1 was added to the media. Cells were prepared for FACS and analysis was performed as described before. Analysis was performed using FlowJo software (FlowJo). FACS was also used to sort cells double transduced for GCaMP6s and Ftractin-mRuby3, following the same protocol.

Generation of KO ESCs Lines

Knock-out ESCs lines for LRP5, LRP6, LRP5/6 and DVL2 were generated by CRISPR/Cas9 mediated gene editing. gRNAs targeting an early exon conserved in all transcriptional variants of each gene were designed using GENEIOUS (Biomatters Ltd.; **Fig. S6**) and scored by their off-target score calculated against the whole mouse genome (7). Oligos coding for the selected gRNAs where sub-cloned into the pSpCas9(BB)-2A-GFP (PX458) plasmid as described elsewhere (8), and the final product was verified by Sanger sequencing. WT W4 ESCs were transiently transfected with the editing plasmids using JetPrime (Polyplus-transfection), as recommended by the manufacturer. 24h post-transfection, cells where trypsinized and GFP^{+} ESCs where FACS-sorted to single cells in 96-well plates. Clonal populations were expanded by splitting the single-cell generated colonies. Gene editing was assessed by PCR amplification of the targeted region, TOPO-cloning of the PCR fragments and Sanger sequencing of at least 12 TOPO-clones. Clonal lines with homozygous mutations that generated a frame-shift mutation inducing a non-functional protein were expanded in Serum + LIF + 2i (9, 10), then the media was changed to Serum + LIF for further characterization and use in the experiments.

Generation of synthetic embryo-like structures

Synthetic embryo like structures were generated as described by Harrison et al (11). Briefly, ESCs and TSCs (constitutively expressing GFP) cultured as described above were trypsinised and dissociated to a single cell suspension (for ESCs) or small clusters of 2-4 cells (for

TSCs). Cells were washed twice with PBS, and 4000 cells/clusters of each type per well were seeded in a matrigel coated IBIDI µ-well glass slide. After 10 min incubation at 37ºC, 5% CO_2 to allow cell attachment, culture media (40% MegaCell RPMI, 25% DMEM/F-12, 25% Neurobasal A supplemented with 10% ES qualified FBS, 2 mM Glutamax, 0.1 mM 2-ME, 0.5 mM sodium pyruvate, 0.25X N2 supplement, 0.5X B27 supplement, 12.5 ng/ml FGF4 and 500 ng/ml heparin) enriched with 10% Matrigel was added to the wells. In some conditions, 2 µM IWP2 or 10 µM CNQX was also added to the media. Synthetic embryo-like structures were cultured for 5 days at 37ºC, 5%CO_2, changing the media daily. For analysis of structure formation, wells were imaged daily for brightfield and GFP channels, on a Zeiss inverted Axio Imager using a 10x objective. An average of 7 representative positions were chosen per condition, replicate and day. Correct embryo-like structure formation was assessed by analyzing the images, according to **Fig. S2**.

Scanning electron microscopy (SEM)

1500 cells were cultured with 0.3 µg Wnt3a beads on a cover slide coated with Fibronectin (10 µg/mL) for 24 hours. The sample was fixed overnight with 2.5% glutaraldehyde in cacodylate buffer at 4ºC. Samples were then post fixed for an hour in 1% Osmiumtetraoxide in cacodylate buffer followed by ethanol stepwise dehydration (50% for 10 min, 70% for 10 min, 80% for 10 min, 95% twice for 5 min and 100% thrice for 5 min), mounting and gold covering. The sample was imaged using a FEI Quanta 200 scanning electron microscope.

Calcium transients measurement

ESCs stably carrying pGP-CMV-GCaMP6s were seeded at a density of 4500 cells per well in imaging plates (ibiTreat µ-Slide 8 well, Ibidi, #80826) and incubated for ≥ 6h in media containing 10% serum and LIF, at 37ºC, 5%CO_2. For ESCs-TSCs Ca^{2+} transient measurements, 4000 ESCs and 4000 TSCs were seeded on an imaging well and incubated together for 6h at 37ºC, 5%CO_2. Cells were transferred to a Nikon Eclipse Ti Inverted Spinning Disk confocal (equipped with a Yokogawa CSU-1 disk head and an Andor Neo sCMOS camera) with an incubation system at 37ºC, 5%CO_2, and beads were added in situ at a concentration of 0.6 µg of beads per well, if required. Cells were further incubated to allow the beads to precipitate for 15 min. Cells in close proximity to beads, or ESCs contacting TSCs were chosen. Images of GCaMP6 (GFP) and DIC on the larger cytonemes were acquired every 6 s for ≈20 min. The generation of localized calcium transients near the bead or TSC at the cytonemes were analyzed. Acquired time-course images where analyzed using FIJI, and the GCaMP6 (GFP) signal was normalized to background.

Quantitative PCR

For transcription analysis of ESCs or TSCs, cells were grown as described, and RNA was extracted using the RNeasy mini kit (Qiagen, #74106). mRNA was retrotranscribed to cDNA using

the QuantiTect Reverse Transcription Kit (Qiagen, #205311). qPCR was performed using TaqMan Fast Advanced Master Mix (ThermoFisher, #444496) and TaqMan probes against Lrp5 (Mm01227476_m1), Lrp6 (Mm00999795_m1), Dvl1 (Mm00438592_m1), Dvl2 (00432899_m1), Dvl3 (Mm00432914_m1) or GAPDH (Mm99999915_g1, ThermoFisher) as described by the manufacturer. Alternatively, qPCR was performed using SYBR™ Green PCR Master Mix and primers against iGluR subunits or Wnt proteins, as previously described. Ct values of targeted genes were normalized to house-keeping gene levels (DCt) and plotted as 2^{-DCt}.

Immunofluorescence

Single cell analysis. ESCs were grown to confluency in complete media containing LIF and 2i (described above). 24 hours prior to use in experimentation, 2i was withdrawn and replaced with soluble Wnt3a protein (100 ng/mL). Cells were trypsinized, neutralized and centrifuged at 1.2 x *g* for 4 minutes. Cells were resuspended in complete media containing LIF and counted to obtain 3000 cells/40 uL media per experimental condition. Cells were combined with 0.3 µg beads/condition with extensive pipetting, and seeded onto human fibronectin (VWR/SLS, #7340101; 10 µg/mL) coated glass 8-well slides (Thistle Scientific, #80828). Cells and beads were incubated at 37ºC, 5%CO_2. Cells were fixed for 8 minutes in 4% paraformaldehyde (PFA) at room temperature (RT), washed 3 times in staining buffer (phosphate buffered saline (PBS, Sigma, d8537) containing 1% BSA (Sigma, #A8806-5G), 0.05% Sodium Azide, and 0.001% Tween20 (Sigma, #P2287-500ML), 5 minutes each) prior to blocking in 2% FBS in staining buffer for 1 hour. Slides were incubated in primary antibody overnight at 4ºC. Following incubation with primary antibody, slides were washed 3 times 5 minutes in staining buffer before incubation with AlexaFluor conjugated secondary antibodies against the appropriate species (Life Technologies) for 1 hour at RT. Final washes were performed with staining buffer containing Hoechst 33342 (Life Technologies, #H3570) at 1:2000 dilution and AF555-Phallodin (ThermoFisher, #A34055) at a dilution to 1:25, before slides were mounted with ProLong mounting media (Life Technologies, #P10144) and coverslipped. Slides were imaged on a Zeiss inverted Axio Imager epifluorescence microscope using Zen 2 (Blue edition) software, 40x (NA = 1.3), equipped with a CoolSNAP HQ2 CCD camera. Alternatively, slides were imaged on a Nikon Eclipse Ti Inverted Spinning Disk confocal (equipped with a Yokogawa CSU-1 disk head and an Andor Neo sCMOS camera) using NIS-Elements. Z-stacks were taken according to the optimal interval, and the range obtained based on β-catenin signal. Images were processed, deconvolved, and quantified by supervised quantification using Volocity software (Perkin Elmer).

Population Analysis: ESCs were grown to confluency in complete media containing LIF and 2i (described above). Cells were trypsinized, neutralized, centrifuged and resuspended in complete media containing LIF and 2i to obtain 5000 cells/200 µL for each condition. Cells were seeded onto

fibronectin-coated glass bottomed black 96 well plates (Greiner CELLSTAR 96 well plates, Sigma, #M0652) and incubated at 37°C, 5%CO_2 for 24 hours. Following incubation, cells were fixed and stained as described above. 96 well plates were imaged using Operetta CLS (Perkin Elmer), taking between 3 and 5 Z-stacks. Images were processed and analyzed in Harmony software (Perkin Elmer).

Cell death was analyzed using the *In situ* cell death detection kit Fluorescein (Roche, #11684795910). Briefly, cells were plated in the same way as described above, fixed for 8 minutes in 4% PFA, permeabilized in staining buffer for 30 minutes and incubated in TUNEL reaction mix for 1 hour at 37°C. Nuclei were labeled by Hoechst at dilution 1:2000 in staining buffer and cells were imaged using the Operetta-Harmony system. Cells undergoing apoptosis were calculated as a percentage of total cell number. Cell proliferation was detected using the Click-iT EdU Alexa Fluor 488 Imaging Kit (Thermo Fisher, #C10337). Cells were plated as described above. After 24 hours, cells were incubated with EdU for 2 hours at 37°C, 5%CO_2. Following this incubation period, cells were fixed, blocked and permeabilized before incubating with Click-iT reaction cocktail for 30 mins RT, protected from light. Cells were washed and nuclei were labeled by Hoechst before cells were imaged using the Operetta-Harmony system (Perkin-Elmer).

Harmony software was used to detect cell nuclei based on DAPI staining. This was used to calculate cell number. ‘Positive’ cells were determined by setting a threshold for GFP fluorescence intensity, and the same threshold was applied to all the experimental conditions.

Western blotting

Cells grown under normal ESC culture conditions were trypsinized and washed in cold PBS prior to lysing with RIPA buffer containing protease and phosphatase inhibitors at 4°C, sonicating for 15 minutes, and centrifuging for 30 minutes at 3200 x *g*. Supernatant was removed and 10 µM phenylmethane sulfonyl fluoride (PMSF, Life Technologies, #36978) added. Protein levels were quantified using the Pierce BCA assay kit (ThermoFisher Scientific, #23225). 10 µg protein was combined with 4X Laemlli Buffer (Bio-Rad, #161-0747) containing 10% β-mercaptoethanol (BioRad, #1610710) and heated to 95°C for 5 minutes prior to loading onto 4-12% stain-free gels (BioRad). Protein transfer was performed using the TransBlot Turbo (BioRad, #1704150), and membranes blocked in 5% milk in TBST (Tris-buffered Saline and 0.1% Tween20) for 1 hour prior to incubation with primary antibody. Membranes were washed 3 times in TBST prior to incubation with horseradish peroxidase (HRP)-conjugated secondary antibody of the appropriate species (Millipore). Bands were visualized via chemiluminesence reaction with Clarity Western ECL detection reagent (BioRad, #1705061) and imaged on the BioRad ChemiDoc Touch (BioRad, #1708370).

Antibodies

The antibodies used were: anti-α-tubulin [YL1/2] (rat; Abcam, ab6160), anti-β-catenin (mouse; BD Transduction, #610154), anti-Dvl2 [30D2] (rabbit; Cell Signalling, #3224), anti-γ-catenin (mouse; BD Transduction, #610253), anti-Lrp6 [EPR2423(2)] (rabbit; Abcam, ab134146), anti-Lrp6 [C5C7] (rabbit; Cell Signalling, #2560S), anti-Nanog (rabbit; Reprocell, RCAB002P-F), anti-Rex1 (rabbit; Abcam, ab28141), anti-Stella (rabbit; Abcam, ab19878), anti-Lrp5 (rabbit; abcam, ab38311) and anti-Cyclophilin B (mouse; R&D systems, #MAB5410).

Karyotyping

At least 2 million confluent ESCs which had had their media replaced no less than 1 hour previously were incubated with fresh media containing 0.02 mg/mL Demecolcine in water (Sigma, D-7385) for 1 hour at 37°C, 5% CO_2 to arrest cells in metaphase. Following incubation, cells were washed twice with sterile PBS prior to trypsinization, neutralization and centrifugation to obtain a pellet. 1 mL KCl was added dropwise to the pellet and agitated by flicking the tube to obtain a single cell suspension. KCl was added to obtain a final volume of 5 mL, and cells incubated 6 mins at RT to swell the cells. Cells were centrifuged at 215 x *g* for 5 minutes, supernatant removed and the cells fixed with 5 mL 3:1 methanol:glacial acetic acid (SLS, #179957-2.5L; Fisher, #10005920) for 5 mins RT. This step was repeated a following 3 times, and the pellet finally resuspended in a final volume of 500μL 3:1 methanol:glacial acetic acid. Cells were dropped from a height of approximately one foot onto ice-cold slides that had been washed in 3:1 methanol: acetic acid. Once dry, slides were immersed in Giemsa stain (Sigma, #GS-500) for 1 hour, washed in water, and imaged on the NanoZoomer slide scanner 2ORS (Hamamatsu). Chromosome number was counted in 300 cells for each cell line and plotted as a histogram.

Statistical analysis and plots

Statistical significance was calculated using Student's T-test (two populations), two-way ANOVA tests (multiple comparisons in more than two groups) or Fisher's exact test (frequencies) using Prism (GraphPad software), as indicated in the figure legends. We used custom Matlab scripts to generate the violin plots. In all tests, statistical significance was quantified as *$p < 0.05$, **$p < 0.01$, ***$p < 0.001$, and ****$p < 0.0001$. We set the threshold for significance as $p < 0.05$, unless specified otherwise.

Figure S1

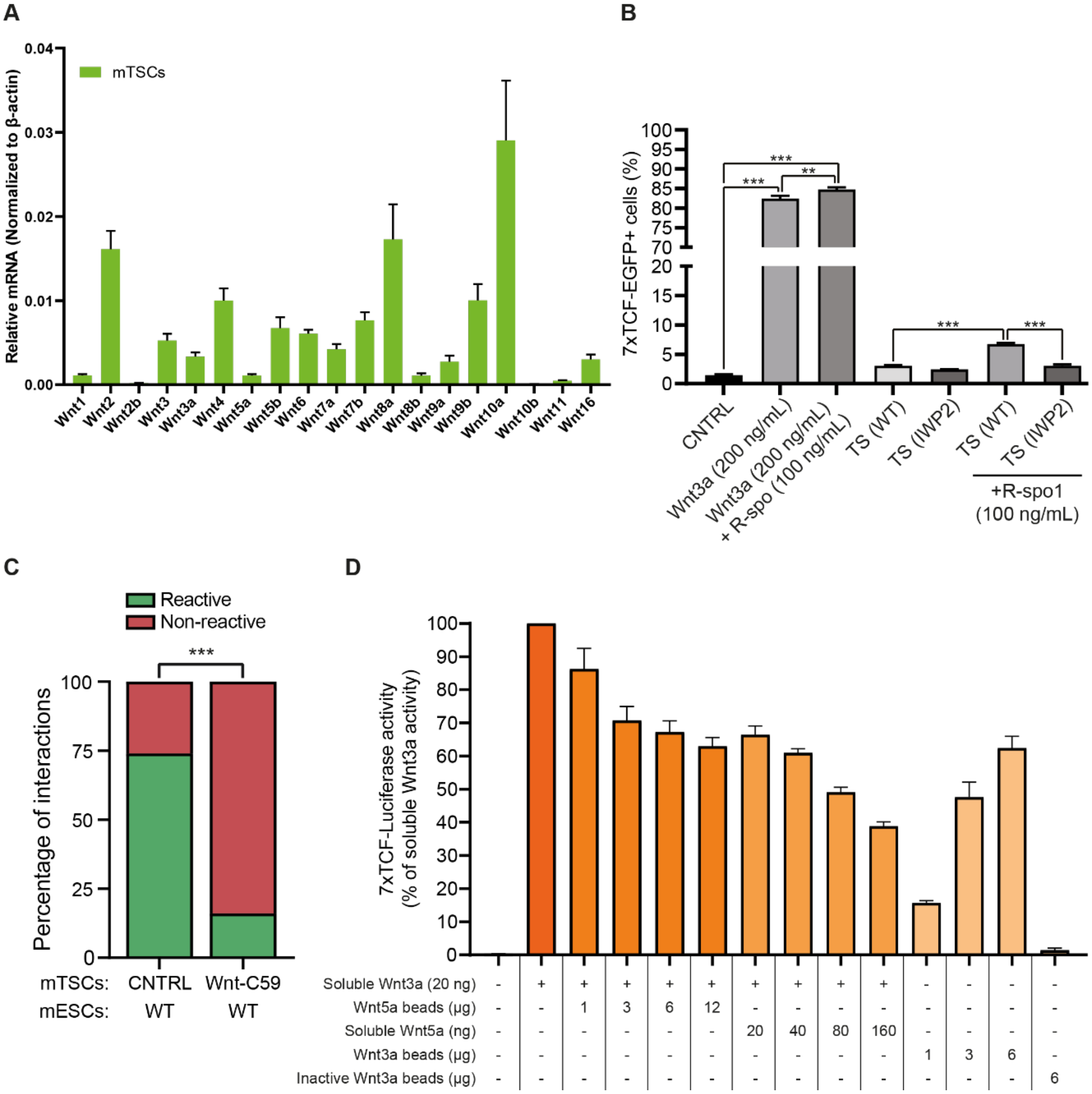

Fig. S1. TSCs produce Wnts that can activate the Wnt/β-catenin pathway in ESCs. A. Transcriptional profile of TSCs regarding the expression of different Wnt proteins. RNA expression is shown as normalized value to β-actin expression. Error-bars represent SEM. n = 3. **B.** Quantification of 7xTCF-eGFP+ ESC cells upon treatment with soluble Wnt3a (200 ng/mL) or upon co-culture with TSCs for 24h, as measured by FACS. IWP2-TSCs were pre-treated with 2 µM IWP2 for 24h before the experiment. R-spondin1 (100 ng/mL) was added to enhance the Wnt pathway activation in some conditions, as labelled. Stars indicate statistical significance calculated by one-way ANOVA test, as follows: ***p<0.01*; ****p<0.001*. **C.** Quantification of reactive (green) or non-reactive (red) interactions between WT ESCs and either CNTRL TSCs or TSCs

treated with 1 μM Wnt-C59 (Porcupine inhibitor) for 24h prior to experiment. N ≥ 44, from 3 independent experiments. Stars indicate statistical significance calculated by Fisher's exact test, as follows: ***$p<0.001$. **D.** Quantification of a representative LSL assay to measure the activity of the immobilized Wnts. Plot represents the percentage of activity of each condition *versus* the Soluble Wnt3a (20 ng) only condition. N = 3, error bars are SEM.

Figure S2

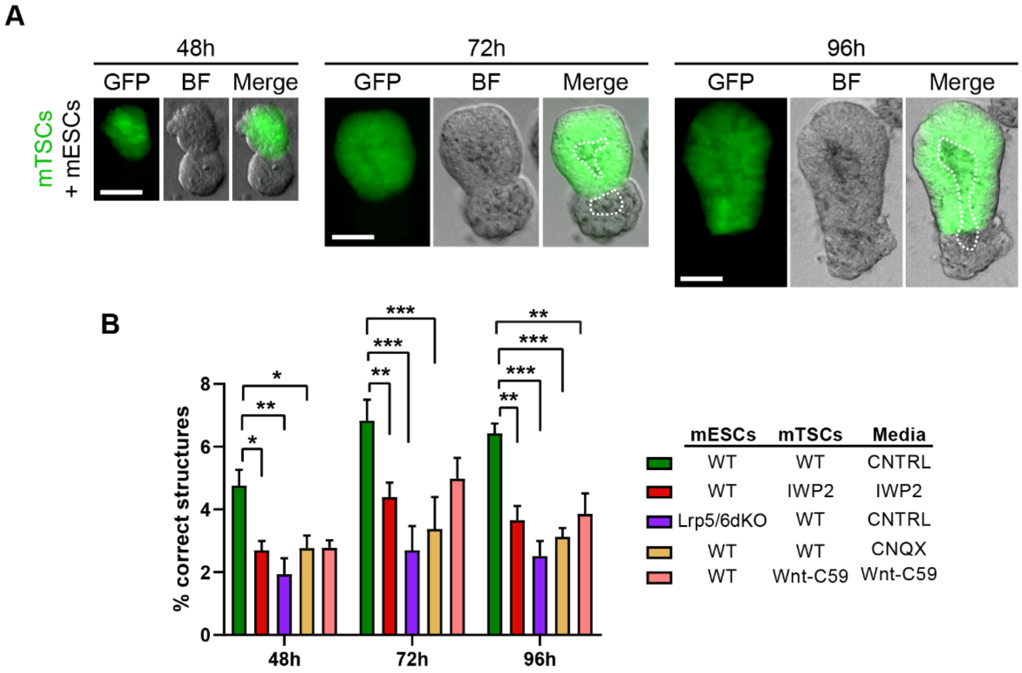

Fig S2. Glutamate receptor activity and Lrp5/6 regulate the formation of synthetic embryo-like structures in 3D culture. A. Representative images of correct synthetic embryo-like structures at different stages (11): at 48h two opposing TSC (constitutively expressing eGFP) and ESC aggregates are contacting; at 72h, the two structures initiate independent cavitation, and at 96h a common cavity is formed along the structure. Dashed white lines show the cavity. Scalebars are 50 μm for all panels. **B.** Bar plot depicting the quantification of correct embryo-like structure formation, quantified according to A. $n \geq 3$, N>100 aggregates/structures per condition per day in technical duplicate. Stars indicate statistical significance as calculated by Two-way ANOVA multiple comparison test, as follows: *$p < 0.05$; **$p < 0.01$; ***$p < 0.001$.

Figure S3

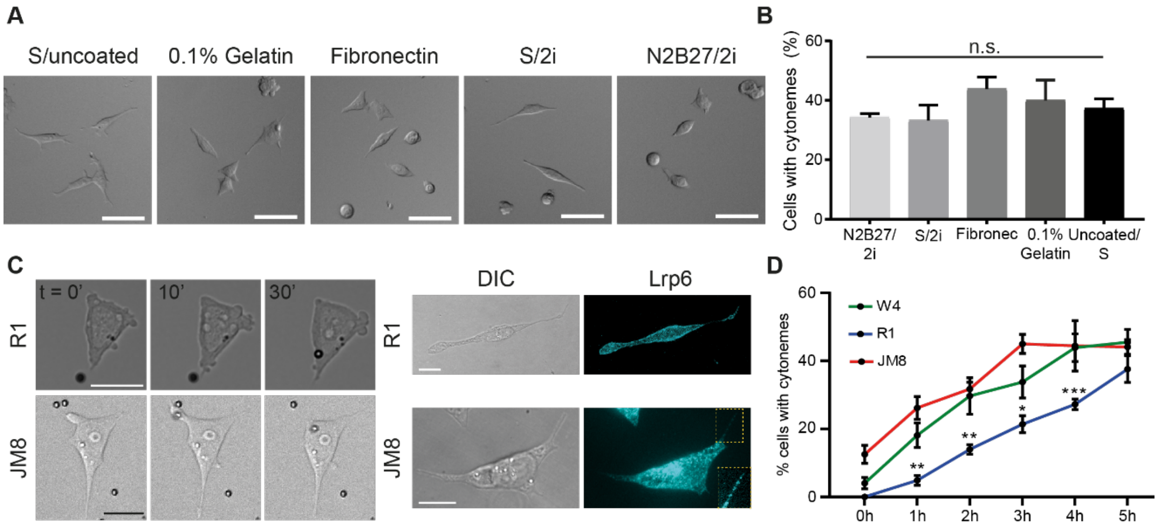

Fig. S3. The presence of ESC cytonemes is independent of substrate, media or genetic background. A. Representative images of ESCs generating cytonemes in various culturing conditions that maintain the naïve state. Scale bars = 20 µm. ESCs were seeded at single cell density on uncoated, 0.1% Gelatin- or 15 µg/mL Fibronectin-coated imaging wells, or on uncoated wells in Serum (S, 5% FBS + LIF), Serum/2i (S/2i, 5% FBS + LIF + 2i) or serum-free N2B27 + 2i media (N2B27/2i + LIF). The presence of cytonemes was monitored by time-lapse imaging for 12h. **B.** Quantification of the percentage of cells generating cytonemes (n = 3, N = 100 cells; statistical non-significance was validated using two-way ANOVA tests). **C.** Representative frames from time-lapse imaging of R1 or JM8 ESCs recruiting a Wnt3a-bead via a cytoneme (left), and representative images of R1 and JM8 cells labelled with antibodies against LRP6 (right). Inset is a contrast-enhanced magnification of yellow box. Scale bars = 10 µm. **D.** Quantification of the percentage of cells with cytonemes in W4, R1 and JM8 cells (n = 3, N = 100 cells; statistical significance is calculated by one-way ANOVA). Stars indicate statistical significance validated by one-way ANOVA test as follows: *$p < 0.05$; **$p < 0.01$; ***$p < 0.001$.

Figure S4

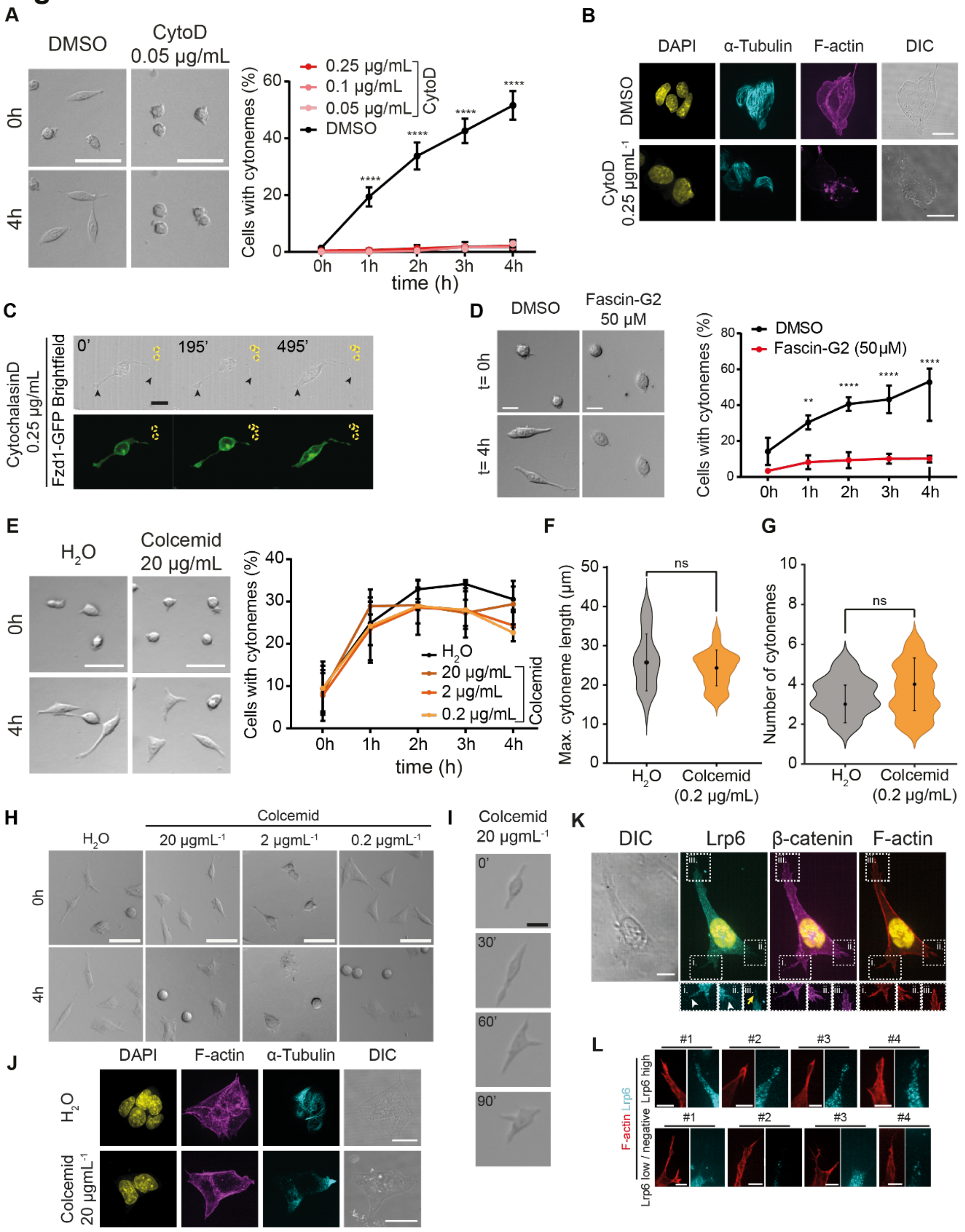

Fig. S4. ESC cytonemes require F-actin for their formation and dynamics and contain components of the Wnt/β-catenin pathway. A. Panels: Representative images of ESCs treated with DMSO or Cytochalasin D and imaged for 4h, scale bar = 20 µm. Graph: Quantification of cells

with cytonemes at different time points (0h to 4h) with DMSO treatment or different concentrations of Cytochalasin D (0.05 to 0.25 µg mL^{-1}). **B.** Cytochalasin D (CytoD, F-actin polymerization inhibitor) effectively depolymerizes actin filaments in an ESC colony. A representative colony of ESC that was treated with 0.25 µg mL^{-1} CytoD or DMSO for 4h, fixed and stained with antibodies for α-tubulin or with AF555-Phalloidin for F-actin. Scale bars = 20 µm. **C.** F-actin inhibition prevents ESC cytoneme formation and reactivity to Wnt3a beads, but existing ESC cytonemes are maintained. Adherent ESCs expressing Fzd1-GFP were treated with 0.25 µg mL^{-1} Cytochalasin D (CytoD) and presented with Wnt3a-beads (dashed yellow circles). Representative images of a CytoD treated ESC exhibiting non-dynamic cytonemes that fail to contact nearby Wnt3a-beads. Arrowheads indicate the tips of the cytonemes. Scale bar = 10 µm. **D**. Actin bundling is required for the formation of ESC cytonemes. ESCs were seeded at a single cell density in the presence of DMSO or 50 µM Fascin-G2 and the generation of cytonemes was monitored by time-lapse imaging for 4h. Panels: representative images. Graph: Quantification of the percentage of the cells with cytonemes in both treatments. n = 3, N > 100 cells. Error bars represent SEM. Stars indicate statistical significance validated by two-way ANOVA test. **E.** Panels: Representative images of ESCs treated with H_2O or Colcemid at 0h or 4h, scale bar = 20 µm. Graph: Quantification of cells with cytonemes at different time points (0h to 4h) treated with control vehicle (H2O) or different concentrations of Colcemid (0.2 to 20 µg mL^{-1}). For **A** and **E**: n=3, N>100 cells. Stars indicate statistical significance validated by two-way ANOVA test. **F.** Violin plots of the quantification of the maximum cytoneme length in ESCs treated with or without Colcemid. **G.** Violin plots of the number of cytonemes in ESCs treated with or without Colcemid. For **F** and **G**, n = 30/condition. Error bars represent SEM. Stars indicate statistical significance validated by Student T-tests. For all experiments: ns, non-significant; *$p<0.05$; **$p<0.01$; ***$p<0.001$; ****$p<0.0001$. **H**. Depolymerization of Tubulin filaments does not affect existing cytonemes. ESCs were allowed to attach and generate cytonemes for 4 hours prior to treatment with H_2O or a range of concentrations of Colcemid. Representative images of ESCs after 4 hours of treatment. Scale bars = 20 µm. **I.** Representative frames from time-lapse imaging showing a 20 µgmL^{-1} Colcemid treated cell that maintains dynamic cytonemes and can form new ones. Scale bar = 20 µm. **J.** Colcemid inhibits tubulin polymerization. ESC colonies were treated with H_2O or 20 µgmL^{-1} Colcemid for 4h, fixed and stained with antibodies for α-tubulin (cyan) or with AF555-Phalloidin for F-actin (magenta). Scale bars = 20 µm. **K.** ESCs cytonemes contain components of the Wnt/β-catenin pathway. Representative ESCs labelled with antibodies against Lrp6 (cyan) and β-catenin (magenta), and counterstained with DAPI and Phalloidin (F-actin, red). Boxes are magnifications of indicated details. White arrowheads indicate Lrp6 high/positive protrusions, yellow arrow indicates Lrp6 low/negative protrusion. Scale bar = 20 µm. 58.9% of cytonemes contain Lrp6 (n = 39 cells). **L.** Multiple examples of ESCs cytonemes stained with

antibodies against Lrp6 (cyan) or Phalloidin (red) representing Lrp6-high/positive cytonemes (top), or Lrp6-low/negative cytonemes (bottom). Scale bars = 10 μm.

Figure S5

A

Cell line	WT (n = 18)	WT (n = 17)	Lrp5/6KO (n = 14)	CNQX (n = 16)	MK801 (n = 17)	EGTA (n = 13)
Bead type	Wnt3a	Inactive Wnt3a	Wnt3a	Wnt3a	Wnt3a	Wnt3a
Cells with Ca^{2+} transients (%)	72.2	23.5	35.7	18.7	52.9	23.1
Mean # transients per 20 min	4.60	2.26	3.03	2.29	4.60	2.63
Mean duration of transients (s)	78.2	31.7	42.2	21.8	83.2	26.6

B

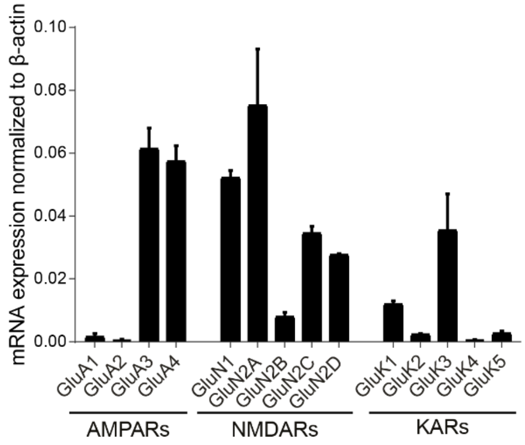

C

Cell line	mESCs	mESCs
Bead type	Wnt3a	Wnt3a
Treatment	Kainate	CNQX+ Kainate
Cells with Ca^{2+} transients	15/18	8/13
Mean # transients/20 min	4.15	2.43
Mean duration transients (s)	24.3	31.7

D

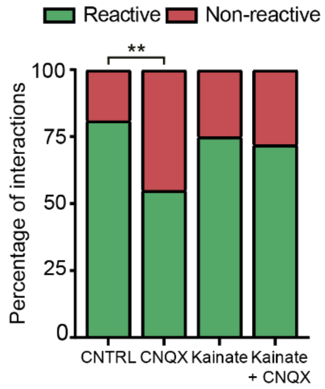

E

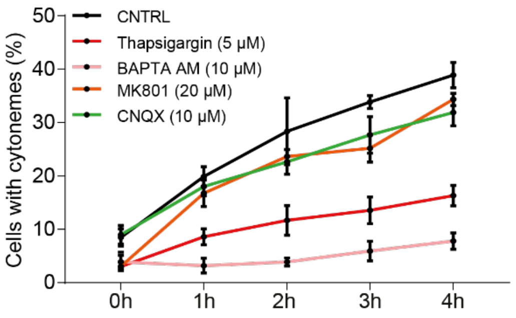

CNTRL vs	0h	1h	2h	3h	4h
BAPTA AM	ns	****	****	****	****
Thapsigargin	ns	**	****	****	****
MK801	ns	ns	ns	ns	ns
CNQX	ns	ns	ns	ns	ns

F

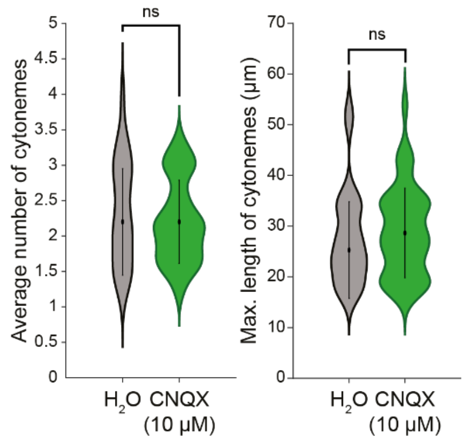

Fig S5. Ca^{2+} transients in ESC cytonemes are dependent on iGluR activity. Cytoneme formation is dependent on intracellular Ca^{2+}. A. Summary table of the quantification of Ca^{2+} transients in the protrusions of ESCs in different conditions (listed in the figure). Abbreviations: percentage (%), number (#), seconds (s). **B.** Transcriptional profile of ESCs regarding the expression of ionotropic GluR subunits, grouped by AMPA-receptors (AMPARs), NMDA-receptors

(NMDARs) or Kainate-receptors (KARs). RNA levels are presented relative to β-actin expression. n = 3, error-bars are SD. **C.** Summary table of the characterization of Ca^{2+} transients in WT ESCs treated with Kainate (100 μM) or CNQX (10 μM) + Kainate (100 μM). Abbreviations: number of cells (n), seconds (s), number (#). **D.** Quantification of the percentage of reactive (green) or non-reactive (red) interactions between ESCs and Wnt3a-beads when treated with CNQX (10 μM), Kainate (100 μM), CNQX + Kainate or H_2O. N ≥ 30, from 3 independent experiments. Data for control (CNTRL) is repeated from **Figure 3D** for comparison. Stars indicate statistical significance calculated by Fisher's exact test, as follows: $^{**}p < 0.01$. **E.** Graph (top) and statistics (bottom) of the percentage of cells with cytonemes in WT cells treated with different conditions or control solution and imaged for 4h. n = 3, N > 30 cells. Stars represent statistical significance validated by two-way ANOVA test as follows: $^{**}p < 0.01$, $^{****}p < 0.0001$. **F.** Violin plots describing the distribution of the average number of cytonemes and the maximum length of cytonemes over the 4h period, in WT cells treated with CNQX (10 μM). N = 42 cells.

Figure S6

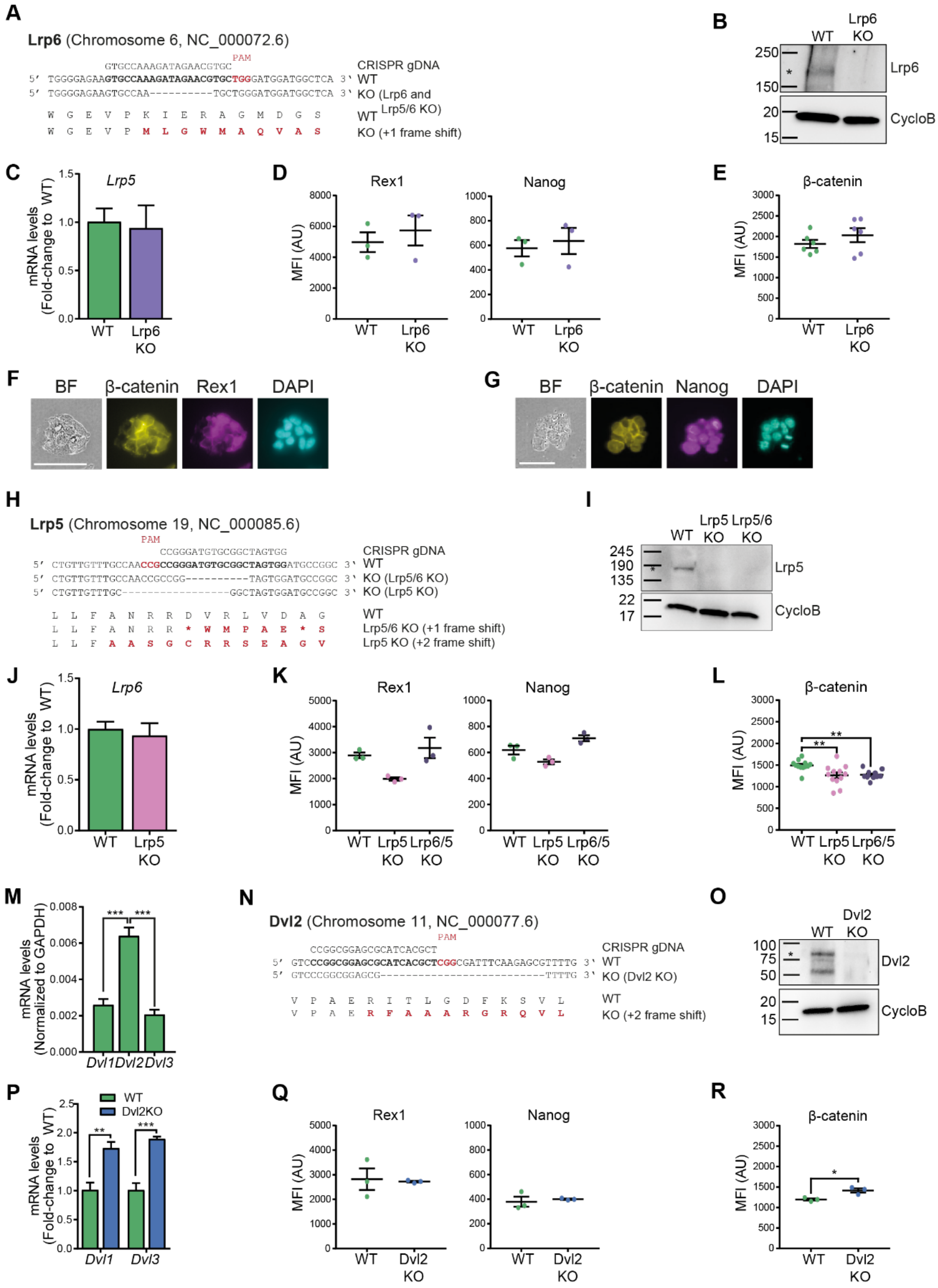

A
Lrp6 (Chromosome 6, NC_000072.6)
CRISPR gDNA
WT
KO (Lrp6 and Lrp5/6 KO)
WT
KO (+1 frame shift)
B
WT
Lrp6 KO
Lrp6
CycloB
C
Lrp5
mRNA levels (Fold-change to WT)
D
Rex1
Nanog
MFI (AU)
E
β-catenin
F
BF
β-catenin
Rex1
DAPI
G
Nanog
H
Lrp5 (Chromosome 19, NC_000085.6)
KO (Lrp5/6 KO)
KO (Lrp5 KO)
Lrp5/6 KO (+1 frame shift)
Lrp5 KO (+2 frame shift)
I
Lrp5 KO
Lrp5/6 KO
Lrp5
J
Lrp6
K
Lrp6/5 KO
L
M
mRNA levels (Normalized to GAPDH)
Dvl1
Dvl2
Dvl3
N
Dvl2 (Chromosome 11, NC_000077.6)
KO (Dvl2 KO)
KO (+2 frame shift)
O
Dvl2 KO
Dvl2
P
Dvl2KO
Q
R

Fig. S6. Generation and characterization of knock out (KO) ESC cell lines. A. The design to generate LRP6KO: Upper panel represents the CRISPR gDNA that targeted the wild type (WT) LRP6 gene DNA of ESCs to generate the KO. Sanger sequencing was used to confirm LRP6KO (KO sequence). The lower panel indicates a portion of the protein sequence of LRP6 in WT cells and the frame shift that generated LRP6KO. **B.** Western blot for WT and LRP6KO ESCs using anti-LRP6 antibody. Cyclophilin B (CycloB) is used as a loading control. **C.** The levels of *Lrp5* mRNA in WT and LRP6KO ESCs. **D** and **E.** Quantification of the mean intensity fluorescence (MFI) of Rex1, Nanog (**D**) and β-catenin (**E)** in WT and LRP6KO. n≥3, N≥30 cells/n. AU: arbitrary units. **F** and **G.** Representative images of quantified colonies. The immunofluorescent staining is as indicated above each image. BF: Bright Field. Scale bar = 50 µm. **H.** The design to generate LRP5KO and LRP5/6dKO: Upper panel represents the CRISPR gDNA that targeted the WT LRP5 gene DNA of WT and LRP6KO ESCs to generate the LRP5KO and LRP5/6dKO, respectively. Sanger sequencing was used to confirm LRP5KO (KO sequences). The lower panel shows a portion of the protein sequence of LRP5 in WT cells and the frame shift generated LRP5KO and LRP5/6dKO (lower line). **I.** Western blot for WT, LRP5KO and Lrp5/6KO ESCs using anti-LRP5 antibody. Cyclophilin B (CycloB) is used as a loading control. **J.** The levels of *Lrp6* mRNA in WT and LRP5KO ESCs. **K** and **L.** Quantification of the mean intensity fluorescence (MFI) of Rex1, Nanog (**K**) and β-catenin **(L)** in WT, LRP5KO and LRP5/6dKO. n≥3, N≥30 cells/n. AU: arbitrary units. **M.** The mRNA levels of DVL1, 2 and 3 in WT ESCs. **N.** The design to generate DVL2KO: Upper panel represents the CRISPR gDNA that targeted the WT DVL2 gene DNA of ESCs to generate the DVL2KO, underneath the DVL2 gene DNA. Sanger sequencing has confirmed DVL2KO (KO sequences). The lower panel shows a portion of the protein sequence of the DVL2 in WT cells and the frame shift that generated DVL2KO (lower line). **O.** Western blot for WT and DVL2KO ESCs using anti-DVL2 antibody. The asterisk indicates the expected molecular weight of DVL2. Cyclophilin B (CycloB) is used as a loading control. **P.** The levels of *Dvl1 and 3* mRNA in WT and DVL2KO ESCs. **Q** and **R.** Quantification of the mean intensity fluorescence (MFI) of Rex1, Nanog (**Q**) and β-catenin **(R)** in WT, DVL2KO and n≥3, N≥30 cells/n. AU: arbitrary units. Stars in dot-plots indicate statistical significance calculated by two-way ANOVA tests, as follow: *p < 0.05; **p < 0.01; ***p < 0.001; ****p < 0.0001.

Figure S7

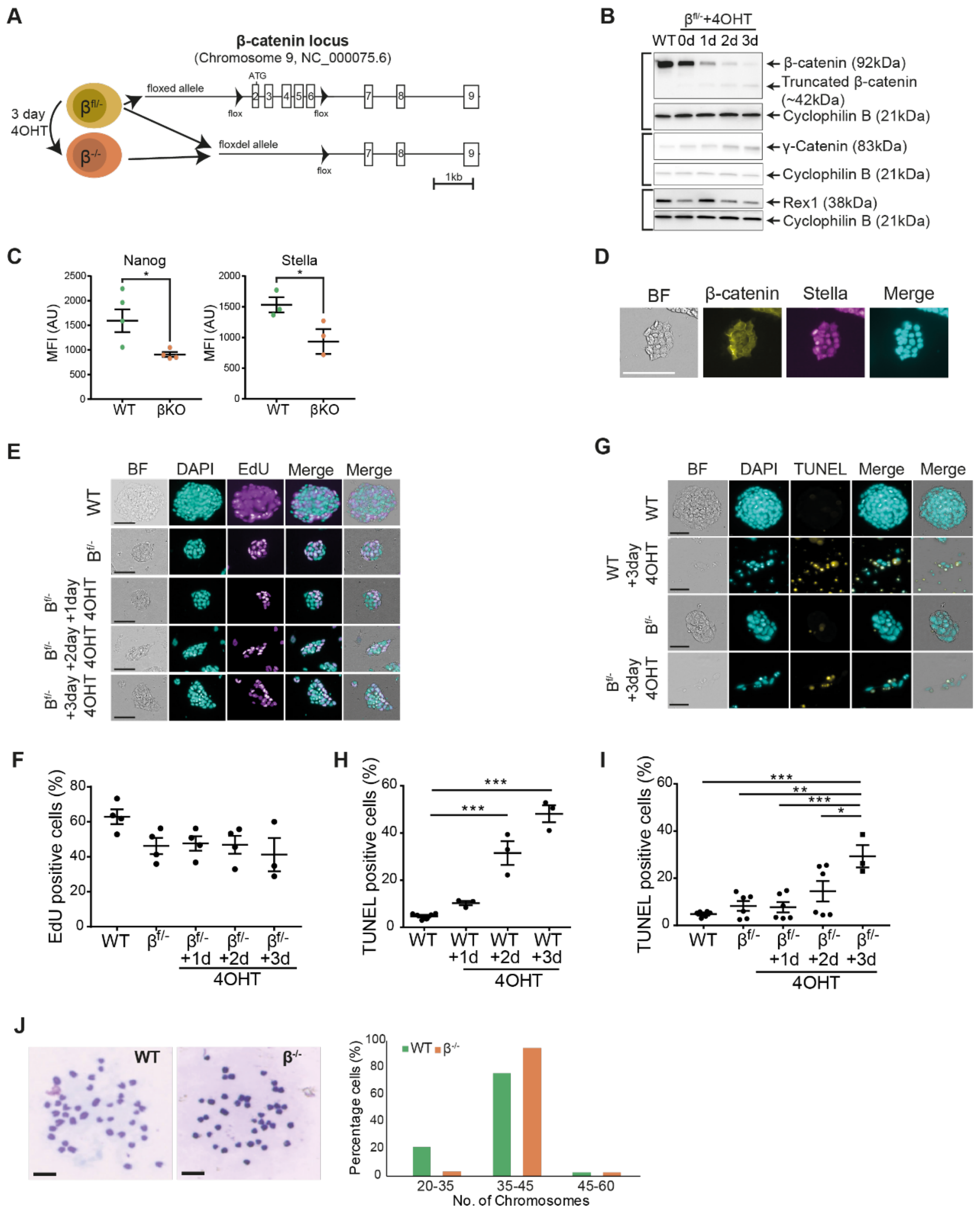

Fig. S7. Characterization of cells containing truncated β-catenin. A. Schematic indicating the allelic variants of the β-catenin-truncated cell line prior to ($\beta^{f/-}$) and after ($\beta^{-/-}$) three days treatment with 4-hydroxy-tamoxifen (4OHT). **B.** Representative β-catenin protein expression in WT, $\beta^{f/-}$ + 0, 1, 2 and 3 days treatment with 4OHT and long term truncated $\beta^{-/-}$ cells, as detected by Western

blotting. A ≈42 kDa truncated form of β-catenin can still be detected. Cyclophilin B is used as loading control. Black brackets (left) indicate same-gel combinations. Cyclophilin B is used as loading control. **C.** Quantification of the mean intensity fluorescence (MFI) of Rex1 and Stella in WT and βKO. n≥3, N≥30 cells/n. AU: arbitrary units. **D**. Representative images of the quantified colonies. The antibodies used for staining are indicated above each image. BF: Bright Field. Scale bar: 50 µm. **E** and **F.** Representative images (**E**) and quantification (**F**) of EdU staining in WT and $\beta^{f/-}$ + 0, 1, 2 and 3 days treatment with 4OHT ESC. **G.** Representative images of TUNEL positive cells (cyan) and DAPI (yellow).**H** and **I**. quantification of TUNEL positive cells in WT and $\beta^{f/-}$ cells treated for 0, 1, 2 and 3 days with 4OHT. **J.** Representative images (left panel) and quantification (right) of karyotyping in WT and $\beta^{-/-}$ cells. Stars in dot-plots indicate statistical significance calculated by two-way ANOVA tests, as follow: *$p < 0.05$; **$p < 0.01$; ***$p < 0.001$; ****$p < 0.0001$.

Figure S8

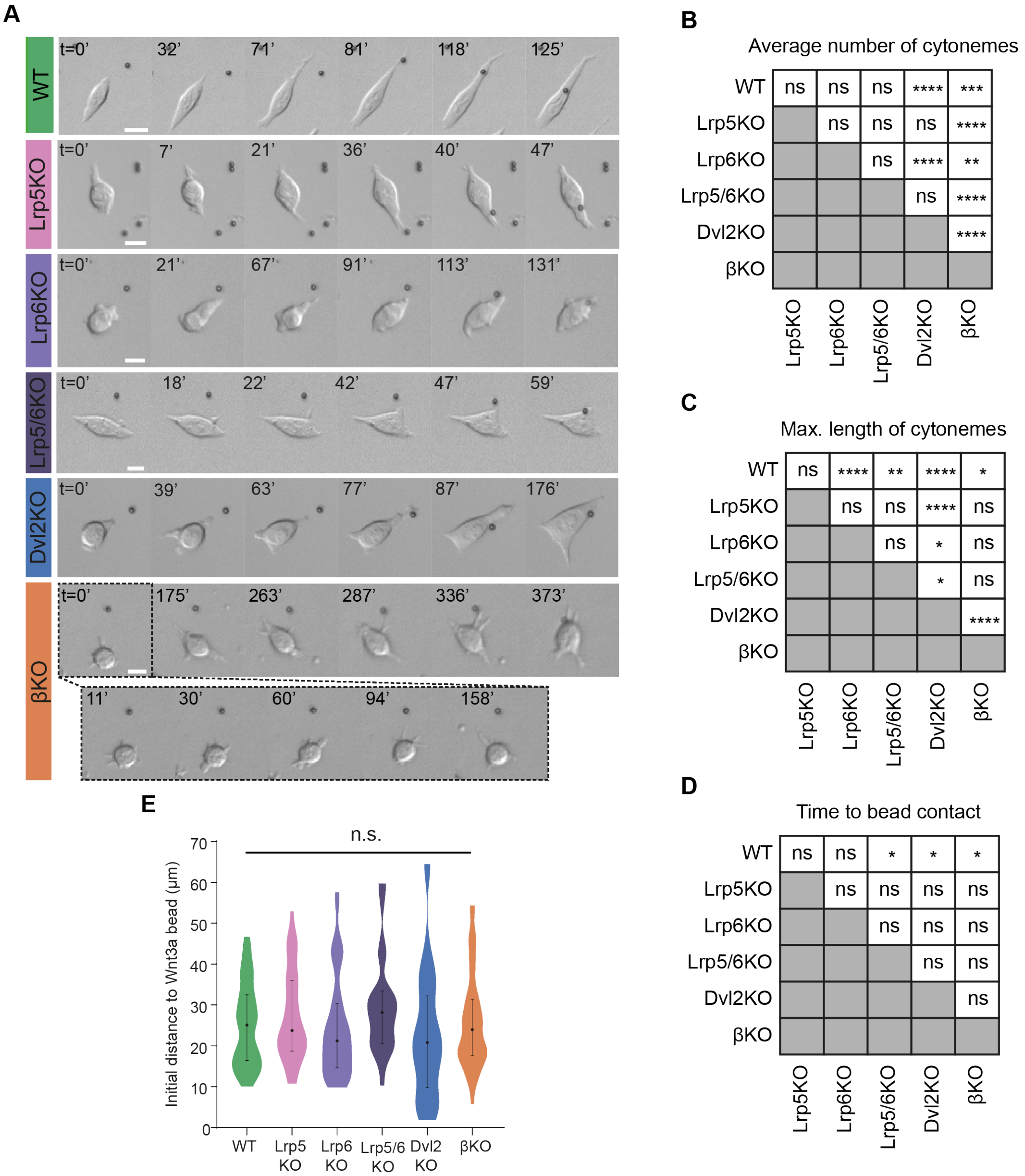

Fig. S8. Characterization of Wnt response and protrusion dynamics in KO cell lines of components of the Wnt pathway. A. Representative time frames of time-lapse imaging showing the dynamics of the cytonemes in WT (top row) and KO ESCs in the presence of Wnt3a-beads (black spheres). Inset in βKO cells shows the specific behaviour of the cytonemes in these cells. Time in minutes. Scale bar = 20 µm. **B**, **C and D.** Summary box showing the statistical significance between the different cell lines in average number of cytonemes (**B**), maximum cytoneme length per cell (**C**) and time to bead contact (**D**), calculated by two-way ANOVA test. For all experiments, stars indicate statistical significance as follows: ns (not significant), **p* < 0.05; ***p* < 0.01; ****p* <

0.001; ****$p < 0.0001$. **E**. Violin plot describing the initial distance between the centre of the cell and the bead for WT and KO ESCs. N > 40 cells from 3 independent experiments. ns is non-significant.

Captions for Movies 1-8 (separate files).

Movie 1. Reactive ESC-TSC interaction. Maximum intensity projection from confocal Z-stack of an ESC (magenta) contacting and pairing with a TSC (green) (reactive interaction). Scale bar is 20 μm.

Movie 2. Non-reactive ESC-TSC interaction. Maximum intensity projection from confocal Z-stack of an ESC (magenta) contacting a TSC (green) but not reacting or pairing with it (non-reactive interaction). Scale bar is 20 μm.

Movie 3. WT mESCs interacting with a Wnt3a bead. High-quality phase images of a WT mESCs contacting a Wnt3a bead through a protrusion, recruiting it to the cell body and orienting the angle of division. Images were obtained by 3D hologram tomography. Scale bar is 20 μm.

Movie 4. Protrusions of WT mESC. High-quality phase images of WT mESCs generating protrusions. Images were obtained by 3D hologram tomography. Scale bar is 20 μm.

Movie 5. Protrusions of LRP5KO mESC. High-quality phase images of LRP5KO mESCs generating protrusions. Images were obtained by 3D hologram tomography. Scale bar is 20 μm.

Movie 6. Protrusions of LRP6KO mESC. High-quality phase images of LRP6KO mESCs generating protrusions. Images were obtained by 3D hologram tomography. Scale bar is 20 μm.

Movie 7. Protrusions of DVL2KO mESC. High-quality phase images of DVL2KO mESCs generating protrusions. Images were obtained by 3D hologram tomography. Scale bar is 20 μm.

Movie 8. Protrusions of βKO mESC. High-quality phase images of βKO mESCs generating protrusions. Images were obtained by 3D hologram tomography. Scale bar is 20 μm.

Supplementary text:

Generation and characterization of knock-out cell lines (KO) of components of the Wnt pathway

The receptors LRP5 and LRP6 share 71% homology in protein sequence and are both required for the activation of the Wnt/β-catenin pathway (12, 13-16). Knocking-out each receptor individually in ESCs with different genetic backgrounds (17-20) did not affect pluripotency, but a LRP5/6 double knock-out embryo was unable to undergo gastrulation (21).

We knocked-out LRP5 and LRP6 separately in W4 ESCs using a CRISPR/Cas9 based approach, designed to generate indel mutations in the early exons of the Lrp5 and Lrp6 genes. A homozygous sequence frame shift that modified the transcription pattern was assessed by Sanger sequencing (**Fig S6A and H**). We also confirmed the knock-out (KO) by using Western blotting (**Fig S6B and I**). Cells lacking LRP6 did not show compensation by an increase of LRP5 at the transcriptional level and vice versa (**Fig S6C and J**). We also knocked out LRP5 in LRP6KO cells to generate a double knock out LRP5/6 cell line (LRP5/6dKO) (**Fig S6A and H-I**). We characterized hallmarks of pluripotency in the KO cell lines and their response to exogenous Wnt ligands. When cultured in the self-renewal media containing serum, LIF and 2i (10), LRP5KO, LRP6KO and LRP5/6dKO maintain similar levels of the core pluripotency transcription factor Nanog and the naïve pluripotency marker Rex1 compared to wild type (WT) ESCs (**Fig S6D, F, G and K**). While LRP6KO maintains similar levels of β-catenin to WT cells (**Fig S6E**), LRP5KO and LRP5/6dKO have reduced levels of β-catenin (**Fig S6L**). Knocking-out LRP5 or LRP6 compromises the ability of ESCs to respond to exogenous Wnt3a, with a more significant reduction in response in LRP6KO. The response to Wnt3a ligands is abolished in the LRP5/6dKO (**Fig 4A**).

The binding of Wnt3a ligands to the receptors initiates a cascade of intracellular signaling that ultimately results in the activation of transcription. The role of downstream signaling components in recruiting the extrinsic signal remains elusive.

To address this, we selected Dishevelled (DVL) and β-catenin, two major and well-characterized components of the Wnt pathway. ESCs express significantly higher levels of DVL2 in comparison to DVL1 or DVL3 (**Fig S6M**). Therefore, we generated DVL2KO ESCs (**Fig S6N and O**). These ESCs upregulate DVL 1 and 3 upon knocking out of DVL2 (**Fig S6P**), have higher levels of cellular β-catenin (**Fig S6R**) and a stronger response to Wnt ligands than the WT cells (**Fig 4A**). The levels of the pluripotency markers remain similar to WT (**Fig S6Q**).

β-catenin has been widely discussed in the context of ESCs (9, 1, 22-24) and we have previously shown polarized localization in response to Wnt3a-beads (6). To investigate the role of β-catenin in the recognition of localized Wnt3a proteins and the subsequent interpretation of the

signal, we examined a previously described conditional β-catenin knock-out cell line (βKO) compared to WT (1). The $\beta^{f/-}$ cell line contains one null allele and the second allele with two LoxP sites to flox exons 2-6 (**Fig S7A**). An inducible KO can be generated from $\beta^{f/-}$ cells by treating with tamoxifen (4OHT) for 3 days (**Fig S7B**). This cell line is employed to minimize the effects of long-term passaging that might lead to cellular compensation mechanisms due to loss of β-catenin.

We found that floxing this cell line abolishes the main protein, as determined by Western blotting, but also results in the generation of a previously undescribed 42kDa fragment that appears specifically in the floxed cell lines upon treatment with 4OHT (**Fig S7B**). Genomic analysis of the flox design indicates an in-frame mutation to β-catenin that encodes a truncated form of the protein (**Fig S7A**). However, this fragment does not appear to accumulate, as there is no increase in the amount detected over time (*i.e.* between 0 and 3 days 4OHT treatment), implying that it is being degraded. Analysis of this fragment revealed the presence of multiple armadillo repeats and a potential nuclear translocation domain, indicating that it may be capable of initiating a response to Wnt signals. The Wnt/β-catenin reporter assay (7xTCF/eGFP) (4) revealed that cells with depleted full-length β-catenin could not induce the expression of GFP (**Fig. 4A**). Therefore, the truncated β-catenin is not capable of initiating transcription.

We further characterized the cells to exclude secondary effects of the loss of β-catenin. 5-ethynyl-2'-deoxyuridine (EdU) incorporation assay revealed a slight (but not significant) reduction in the proliferation of inducible βKO (**Fig S7E and F**). This apparent decrease in proliferation in βKO is accompanied by an increase in the amount of cell death as measured by TUNEL assay (**Fig S7G and I**). An increased propensity for βKO to undergo apoptosis has previously been attributed to the loss of β-catenin (1). However, our results suggest that tamoxifen treatment may contribute to this increase in cell death, as treatment of WT cells with tamoxifen causes a significant increase in the percentage of TUNEL positive cells (**Fig S7H**). Raggioli et al. (1) report that the loss of β-catenin may also affect chromosome stability since they observed an increased number of βKO with lagging chromosomes during anaphase. We karyotyped both the WT and long-term cultured βKO cells, but we did not find any significant differences in chromosome number (**Fig S7J**) between both cell lines.

Wnt/β-catenin signaling is implicated in the maintenance of pluripotency, while β-catenin itself is also critical for cell-cell adhesion (25). The loss of β-catenin has been reported to affect both functions, despite some studies reporting potential compensation by similar molecules such as γ-catenin (1, 22). We employed quantitative immunofluorescence and Western blotting and observed reduction in the expression of the pluripotency markers Nanog, Rex1 and Stella in βKO cells compared to WT (**Fig S7B-D**). Thus, it appears that the loss of β-catenin may alter the naïve

state of ESCs to more closely resemble cells that are differentiation prone (26, 27). To conclude, the loss of β-catenin alters the cell fate and morphology but not genomic stability, the rate of cell division or cell death. Therefore, βKO is suitable for further elucidating the patterns of cell interaction with niche signals.

SI references

1. Raggioli A, Junghans D, Rudloff S, Kemler R (2014) Beta-catenin is vital for the integrity of mouse embryonic stem cells. *PLoS ONE* 9(1):e86691.

2. Tanaka S, Kunath T, Hadjantonakis AK, Nagy A, Rossant J (1998) Promotion of trophoblast stem cell proliferation by FGF4. *Science* 282(5396):2072–2075.

3. Tanaka S (2006) Derivation and culture of mouse trophoblast stem cells in vitro. *Methods Mol Biol* 329:35–44.

4. Fuerer C, Nusse R (2010) Lentiviral vectors to probe and manipulate the Wnt signaling pathway. *PLoS ONE* 5(2):e9370.

5. Willert KK, et al. (2003) Wnt proteins are lipid-modified and can act as stem cell growth factors. *Nature* 423(6938):448–452.

6. Habib SJ, et al. (2013) A localized Wnt signal orients asymmetric stem cell division in vitro. *Science* 339(6126):1445–1448.

7. Hsu PD, et al. (2013) DNA targeting specificity of RNA-guided Cas9 nucleases. *Nat Biotechnol* 31(9):827–832.

8. Ran FA, et al. (2013) Genome engineering using the CRISPR-Cas9 system. *Nat Protoc* 8(11):2281–2308.

9. Berge ten DD, et al. (2011) Embryonic stem cells require Wnt proteins to prevent differentiation to epiblast stem cells. *Nat Cell Biol* 13(9):1070–1075.

10. Raggioli A, Junghans D, Rudloff S, Kemler R (2014) Beta-catenin is vital for the integrity of mouse embryonic stem cells. *PLoS ONE* 9(1):e86691.

11. Harrison SE, Sozen B, Zernicka-Goetz M (2018) In vitro generation of mouse polarized embryo-like structures from embryonic and trophoblast stem cells. *Nat Protoc* 13(7):1586–1602.

12. Brown SD, et al. (1998) Isolation and Characterization of LRP6, a Novel Member of the Low Density Lipoprotein Receptor Gene Family. *Biochem Biophys Res Commun* 248(3):879–888.

13. Tamai K, et al. (2000) LDL-receptor-related proteins in Wnt signal transduction. *Nature* 407(6803):530–535.

14. Semënov MV, et al. (2001) Head inducer Dickkopf-1 is a ligand for Wnt coreceptor LRP6. *Curr Biol* 11(12):951–961.

15. Schweizer L, Varmus H (2003) Wnt/Wingless signaling through beta-catenin requires the function of both LRP/Arrow and frizzled classes of receptors. *BMC Cell Biol* 4(1):4.

16. MacDonald BT, He X (2012) Frizzled and LRP5/6 receptors for Wnt/β-catenin signaling. *Cold Spring Harb Perspect Biol* 4(12):a007880–a007880.

17. Kato M, et al. (2002) Cbfa1-independent decrease in osteoblast proliferation, osteopenia, and persistent embryonic eye vascularization in mice deficient in Lrp5, a Wnt coreceptor. *J Cell Biol* 157(2):303–314.

18. Gong Y, et al. (2001) LDL receptor-related protein 5 (LRP5) affects bone accrual and eye development. *Cell* 107(4):513–523.

19. Asaba H, et al. (2003) 3P-0746 Low-density lipoprotein receptor-related protein 5 (LRP5) is essential for normal cholesterol metabolism and glucose-induced insulin secretion. *Atherosclerosis Supplements* 4(2):225.

20. Pinson KI, Brennan J, Monkley S, Avery BJ, Skarnes WC (2000) An LDL-receptor-related protein mediates Wnt signalling in mice. *Nature* 407(6803):535–538.

21. Kelly OG (2004) The Wnt co-receptors Lrp5 and Lrp6 are essential for gastrulation in mice. *Development* 131(12):2803–2815.

22. Lyashenko N, et al. (2011) Differential requirement for the dual functions of β-catenin in embryonic stem cell self-renewal and germ layer formation. *Nat Cell Biol* 13(7):753–761.

23. Hoffmeyer K, et al. (2012) Wnt/β-catenin signaling regulates telomerase in stem cells and cancer cells. *Science* 336(6088):1549–1554.

24. Sokol SY (2011) Maintaining embryonic stem cell pluripotency with Wnt signaling. *Development* 138(20):4341–4350.

25. Valenta T, Hausmann G, Basler K (2012) The many faces and functions of β-catenin. *EMBO J* 31(12):2714–2736.

26. Hayashi K, de Sousa Lopes SMC, Tang F, Surani MA (2008) Dynamic Equilibrium and Heterogeneity of Mouse Pluripotent Stem Cells with Distinct Functional and Epigenetic States. *Cell Stem Cell* 3(4):391–401.

27. Toyooka Y, Shimosato D, Murakami K, Takahashi K, Niwa H (2008) Identification and characterization of subpopulations in undifferentiated ES cell culture. *Development* 135(5):909–918.
